# *In Vivo* Ultrasound and Photoacoustic Imaging of Nanoparticle-Engineered T Cells and Post-Treatment Assessment to Guide Adoptive Cell Immunotherapy

**DOI:** 10.1021/acsnano.4c12929

**Published:** 2025-02-05

**Authors:** Kelsey P. Kubelick, Jinhwan Kim, Myeongsoo Kim, Xinyue Huang, Chenxiao Wang, Seoyoon Song, Younan Xia, Stanislav Y. Emelianov

**Affiliations:** † Wallace H. Coulter Department of Biomedical Engineering, Georgia Institute of Technology and Emory University School of Medicine, Atlanta, Georgia 30332, United States; ‡ School of Electrical & Computer Engineering, Georgia Institute of Technology, Atlanta, Georgia 30332, United States; § School of Chemistry and Biochemistry, Georgia Institute of Technology, Atlanta, Georgia 30332, United States; ∥ School of Chemical and Biomolecular Engineering, Georgia Institute of Technology, Atlanta, Georgia 30332, United States

**Keywords:** ultrasound, photoacoustic imaging, nanoparticles, cell surface engineering, T cells, cancer immunotherapy, cell tracking

## Abstract

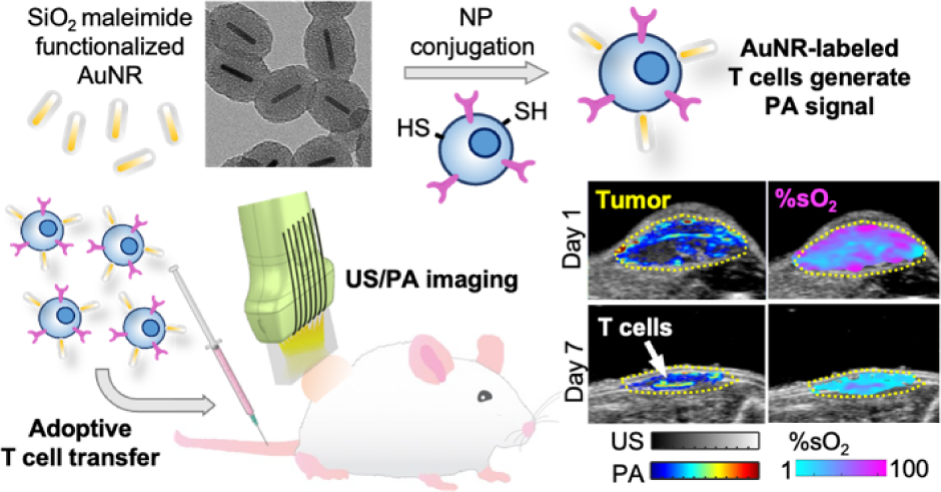

Despite great promise, adoptive cell therapy (ACT) continues to fail at treating a majority of cancers, especially solid tumors. To inform development and expedite the translation of more potent cellular immunotherapies, advanced immunoimaging tools are needed to better understand the *in vivo* requirements for generating a robust immune response. Even methods to evaluate the delivery, location, and status of transferred T cells at the tumor target are lacking. Therefore, a real-time, safe, noninvasive, longitudinal imaging method is critically needed to 1) monitor adoptive T cell location and status and 2) assess treatment progression and response through imaging biomarkers. Here, we developed a combined ultrasound (US) and photoacoustic (PA) imaging approach to enable T cell tracking following adoptive transfer for cancer immunotherapy. Our approach leverages highly photostable gold nanorods and cell surface engineering to tag the T cells without impacting effector functions, as well as generate PA contrast for imaging post-transfer. Our *in vivo* US/PA imaging approach detected nanoparticle-labeled T cell accumulation at the tumor, visualized changes in tumor volume, and conveyed accompanying changes in blood biomarkers. US/PA data also showed different trends according to a positive or negative antitumor response to T cell therapy over 7 days. Results highlight the potential of the approach and motivate future development to expand the platform for advanced, theranostic immunoimaging.

Cancer continues to rank as a leading cause of death worldwide.^1^ A major difficulty in treatment lies in the heterogeneity across patients and cancer types. Personalized treatment methods are rapidly evolving, including paradigm-shifting, targeted immunotherapy.^2^ The goal of immunotherapy is to stimulate and enhance a patient’s immune system to mount a more potent antitumor immune response.^3^ In particular, cell-based immunotherapies have gained momentum, where immune cells are isolated or engineered with receptors that specifically target the patient-specific molecular profile of cancer cells. For example, chimeric antigen receptor (CAR) T cell treatment has a high clinical success rate of 80–90% for the treatment of certain hematological malignancies.^4−8^ To date, six CAR T cell products are on the market for treating blood cancers and lymphoma.^9,10^

In spite of great promise, adoptive cell therapy (ACT), in general, fails to treat a majority of solid tumors, such as breast cancer.^11−14^ To address this problem, better tools are needed to augment cellular immunotherapies by improving our understanding of the *in vivo* requirements for generating a robust immune response.^15−18^ A variety of imaging modalities have been explored to guide adoptive T cell therapies, including X-ray computed tomography (CT), magnetic resonance imaging (MRI), positron emission tomography (PET), single photon emission computed tomography (SPECT), and optical imaging.^19−23^ PET has gained momentum in the immunoimaging space, known as immuno-PET.^24−26^ However, challenges such as ionizing radiation, poor resolution, and high cost are significant barriers to its further translation. Therefore, translation of adoptive cell therapy (ACT) would be expedited by developing a real-time, longitudinal, and safe imaging method to monitor cell location and status while assessing treatment progression through imaging biomarkers.^12,13,19,27−29^

In cell tracking applications, ultrasound (US) and photoacoustic (PA) imaging has the advantages of nonionizing radiation, ease of real-time imaging, relatively low cost, portability, and safety.^30−34^ As a hybrid imaging modality, PA imaging leverages the benefits of light and sound. By irradiating an optical absorber with pulsed laser light, thermal deposition and rapid expansion of the surrounding tissue generates a sounds wave. PA contrast is based on optical absorption, but sound is received to increase imaging depth compared to purely optical methods, achieving imaging depths of 5–7 cm.^35−37^ Therefore, PA imaging enables high contrast, high resolution imaging for functional and molecular assessments at the cellular or tissue level, and US provides critical anatomical context.

PA imaging requires the use of endogenous or exogenous absorbers to enable molecular, cellular, or functional imaging.^30^ Endogenous absorbers, including oxygenated hemoglobin, deoxygenated hemoglobin, and melanin, can be utilized for label-free imaging. Label-free US/PA imaging approaches have been deployed for visualizing changes in blood biomarkers following treatment, including changes in % oxygen saturation to assess hypoxia at the tumor or metastatic lymph nodes.^38−42^ Exogenous contrast agents can be introduced to visualize specific imaging targets. Among the various exogenous contrast agents, gold (Au) nanoparticles (NPs) are particularly favorable due to their customizable spectral signatures, which can be tailored based on composition, size, and shape.^43−49^ Distinct spectral signatures of endogenous and exogenous absorbers can be leveraged for multiplex PA imaging, where multiwavelength sweeps and spectral unmixing can visualize multiple species. Ease of multiplex imaging is a distinct advantage of PA imaging compared to PET, CT, or MRI. US/PA imaging, augmented with NPs, has been deployed for a variety of applications of cell tracking, particularly in the regenerative medicine space.^50−55^ Stem cells labeled with AuNPs can generate PA signals to enable monitoring.^56−61^ To further elucidate the response to cellular therapies, PA signals from oxygenated and deoxygenated hemoglobin can be analyzed to evaluate related changes at the treatment target.

Herein, we report a multiplexing US/PA imaging approach to track adoptively transferred T cells in the context of cancer immunotherapy. The approach leverages highly photostable Au nanorods (AuNRs) and cell surface engineering to tag T cells to generate PA contrast. Following AuNR characterization, PA imageability *in vitro*, and cytocompatibility, we developed a US/PA imaging approach to monitor adoptively transferred NP-labeled T cells *in vivo*. Results highlight the advantages of US/PA imaging for cancer immunotherapies, including multiplex imaging of exogenous and endogenous absorbers. Our findings demonstrate that the US/PA imaging platform enables *in vivo* detection and monitoring of NP-labeled T cells. Accompanying changes in endogenous absorbers were visualized to provide additional feedback on the response to ACT, indicating potential of our US/PA imaging platform to guide and enhance adoptive cell cancer immunotherapy in the future.

## Results and Discussion

**Figure 1:**
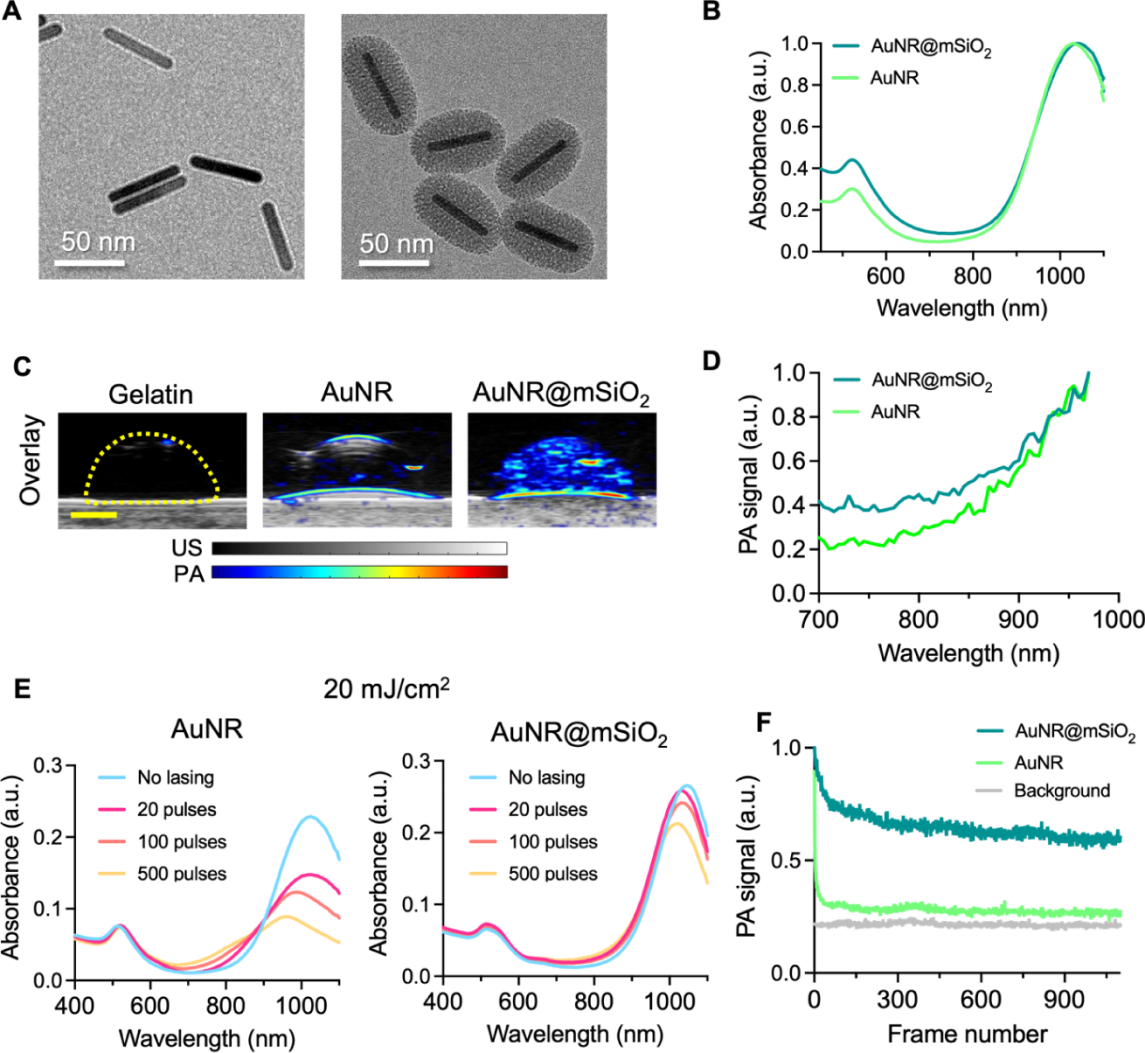
Silica-coated small AuNRs have favorable properties for PA imaging. (A) TEM images of AuNRs before (left) and after (right) silica coating. (B) UV–vis-NIR absorbance spectrum of bare or silica-coated AuNRs, denoted as AuNR (light green line) or AuNR@mSiO_2_ (dark green line), respectively. (C) US (grayscale)/PA (color scale, 1064 nm wavelength) overlay images of a tissue-mimicking phantom, where the dome-shaped inclusions (yellow outline) contained gelatin (background) and AuNRs (middle) or AuNR@mSiO_2_ (right); OD = 5 for both AuNR samples; scale bar = 2 mm. (D) Corresponding PA spectrum of AuNR (light green line) or AuNR@mSiO_2_ (dark green line). (E) UV–vis-NIR absorbance spectrum of AuNR (left) or AuNR@mSiO_2_ (right) samples following continuous pulsed laser irradiation at 1064 nm wavelength highlights improved photostability of AuNR@mSiO_2_, whose optical absorption peak showed little decay up to 500 pulses at 20 mJ/cm^2^ fluence. (F) Average PA signal from AuNR (light green line) or AuNR@mSiO_2_ (dark green line) during continuous pulsed laser irradiation at 1064 nm wavelength also showed enhanced stability from silica coating to enable longitudinal PA imaging.

Contrast agent design is a critical first step toward our goal of developing an imaging approach for longitudinal, sensitive detection of T cells. The ideal contrast agent will efficiently generate strong PA signals with a distinct optical spectrum, is photostable, and can safely label T cells without impacting effector functions. We strategically selected and synthesized silica-coated, small AuNRs with peak optical absorption at 1064 nm as our PA imaging agents to maximize imaging depth and minimize background signals from endogenous absorbers.^46,47^ Transmission electron microscopy (TEM) images verified successful synthesis and the expected morphology of the AuNRs (Figure 1A), in alignment with previous literature.^46,47,62^ Bare, CTAB-stabilized AuNRs had dimensions of roughly 8 nm by 50 nm (Figure 1A; left). To improve photostability^62,63^ the AuNRs were then coated with a mesoporous silica layer (Figure 1A; right). Henceforth, these are referred to interchangeably as “silica-coated AuNRs” or using the abbreviated name of AuNR@mSiO_2_. UV–vis spectrophotometry indicated similar optical absorption of AuNRs before and after silica coating, with an optical absorption peak around 1064 nm (Figure 1B). We proceeded with PA studies in tissue-mimicking gelatin phantoms to confirm imageability. AuNRs and silica-coated AuNRs were prepared in dome-shaped inclusions and imaged at 1064 nm wavelength (Figure 1C) or with multiwavelength spectral scans (Figure 1D). PA signal was observed from both AuNR-containing inclusions. The PA spectral scans from 680–970 nm optical wavelengths agreed with the results from UV–vis spectrophotometry.

The advantage of the silica-coating became apparent upon assessment of photostability.^46,62,64^ Following pulsed laser irradiation at 1064 nm, the bare AuNRs showed substantial decay in optical absorption after only 20 pulses at 8 mJ/cm^2^ (Figure S1) and 20 mJ/cm^2^ (Figure 1E). The silica-coated AuNRs maintained peak optical absorption in both cases, and decay in optical absorption was not observed until reaching 500 pulses at 20 mJ/cm^2^. During continuous PA imaging at 1064 nm wavelength, enhanced stability was observed for the silica-coated AuNRs relative to the bare sample (Figure 1F). Although ∼30% decay was observed for the silica-coated AuNRs, the PA signal stabilized around 300 frames. The PA signal from bare AuNRs rapidly decayed and approached background after only 6 frames (Figure S2A). Importantly, minimal change in the PA spectrum was observed for the silica-coated AuNRs compared to the bare sample after 1000 frames, or 4000 laser pulses, at 1064 nm wavelength (Figure S2B–E). Altogether, the results indicate that the silica-coated small AuNRs achieved the desired optical absorption in the near-infrared (NIR)-II window and produced stable PA signal at 1064 nm. The spectral signatures in the range of 680–970 nm were also stable enough to enable multiwavelength imaging.

**Figure 2:**
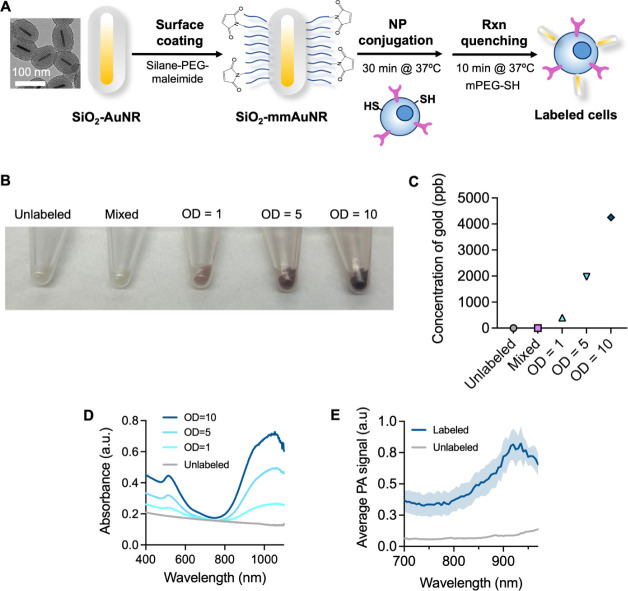
Maleimide functionalization of silica-coated AuNRs enables T cell labeling. (A) Schematic of T cell labeling protocol with maleimide-modified silica-coated AuNRs (SiO_2_-mmAuNR). (B) Photographs of primary murine T cell pellets post labeling, where a darker cell pellet corresponds to increased NP labeling density (OD = 1, 5, and 10, left to right). “Mixed” indicates T cells were coincubated with SiO_2_–AuNR without maleimide functionalization, indicating that maleimide is essential for T cell labeling. (C) ICP-MS to quantify the content of Au per T cell sample. The number of T cells per sample was constant. (D) UV–vis absorbance spectrum of NP-labeled T cells, where an increase in absorbance corresponds to a greater NP labeling density. (E) PA spectrum of NP-labeled T cells (OD = 10 labeling density) and unlabeled T cells from 680–970 nm wavelength at 1000 cells/*μL*.

After demonstrating favorable optical and PA properties of the silica-coated AuNRs, we proceeded to develop an approach for T cell labeling, with an initial focus on Jurkat T cells (Figures S3–S5). The protocol was optimized to maximize PA signals and cytocompatibility. Successful NP-labeling studies in Jurkats informed and motivated all proceeding studies in primary murine T cells. Due to the limited phagocytic activity of T cells to facilitate NP labeling via uptake, cell surface labeling was required to efficiently tag T cells (Figure 2A). The surface of silica-coated AuNRs were functionalized with maleimide, which can then covalently bind to thiols expressed on the T cell surface. Photographs of cell pellets postlabeling showed qualitative differences in labeling efficiency (Figure 2B), where darker cell pellets were observed for T cells incubated with AuNRs at higher optical densities (ODs). The “mixed” condition indicates T cells that were incubated with silica-coated AuNRs without maleimide functionalization. In the mixed case, negligible change in color was observed, indicating that maleimide functionalization was required to label T cells with the AuNRs. Without maleimide, minimal, nonspecific NP/T cell interactions were observed. The Au content in each cell sample was quantified using inductively coupled plasma mass spectrometry (ICP-MS) (Figure 2C). The scanning electron microscopy (SEM) image in Figure S6 clearly showed the AuNRs conjugated on the surface of T cells. Analysis of T cell labeling following treatment with an endocytosis inhibitor provides further evidence of a surface labeling mechanism (Figure S7). Henceforth, the term “NP-labeled T cells” refers to conjugation of maleimide-modified, silica-coated AuNRs to the T cells.

**Figure 3:**
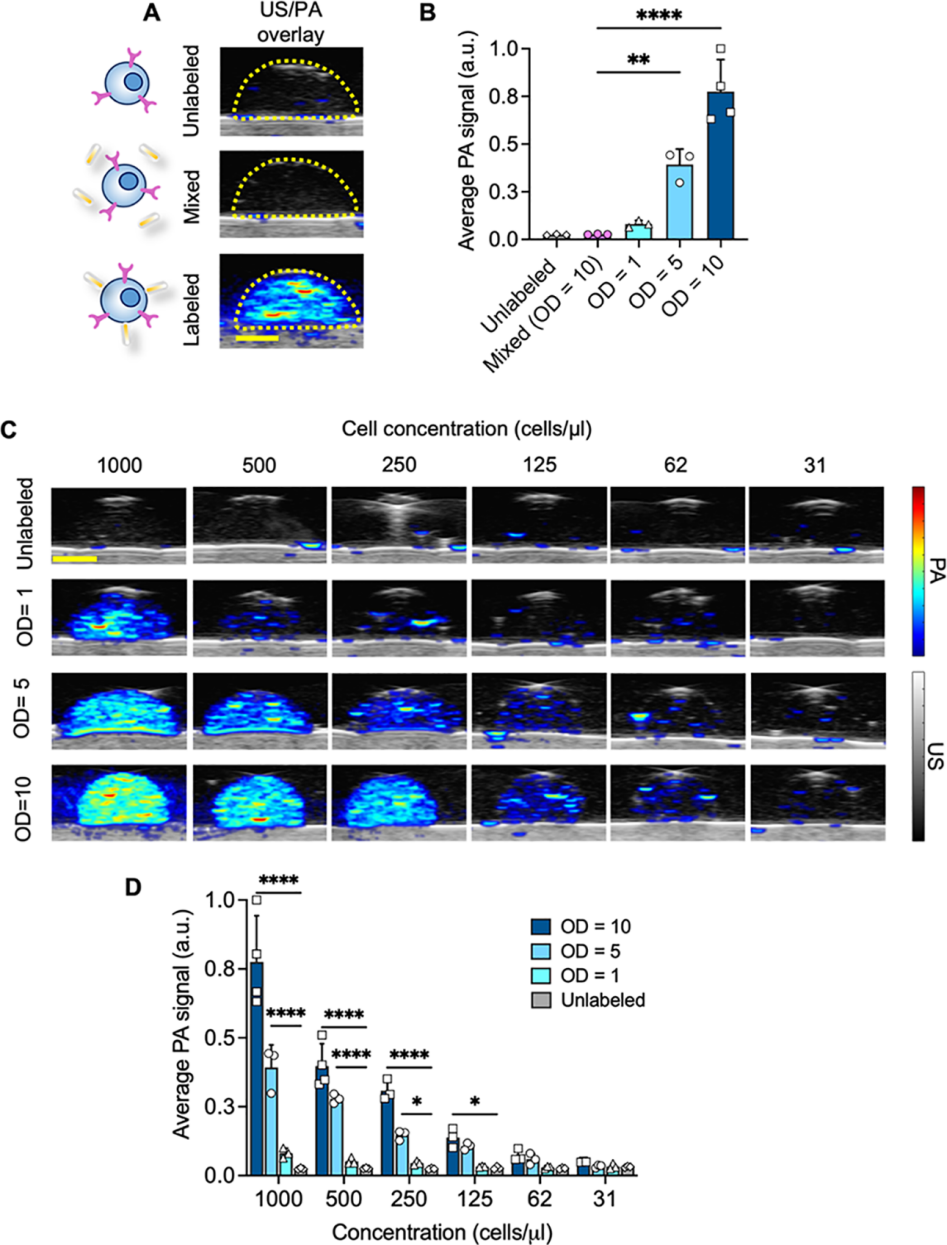
Nanoparticle-labeled T cells generate PA signals *in vitro.* (A) US (grayscale)/PA (color scale, 1064 nm wavelength) overlay images of a tissue-mimicking phantom, where the dome-shaped inclusions (40*μL*) contained the unlabeled control (background), T cells mixed with AuNRs@mSiO_2_ without maleimide functionalization (OD = 10, middle), or T cells labeled with maleimide-modified AuNRs@mSiO_2_ (OD = 10, bottom). All samples were at 1000 cells/*μL.* (B) Corresponding quantitative analysis of (A). Plotted values represent the mean ± standard deviation. Data were analyzed by one-way ANOVA followed by a post hoc Dunnett test. (C) US/PA overlay images and (D) corresponding analysis of a serial dilution phantom of NP-labeled T cells subject to different labeling conditions to assess limits of detection *in vitro*. Scale bar = 2 mm. Plotted values represent the mean ± standard deviation. Data were analyzed by two-way ANOVA followed by a post hoc Dunnett Test. * *p* < 0.05; ** *p* < 0.01; *** *p* < 0.001; **** *p* < 0.0001.

Given the excellent optical properties of the silica-coated AuNRs and successful T cell labeling, we anticipated favorable optical properties and high PA signals from the labeled T cells *in vitro*. UV–vis spectrophotometry showed an absorption peak of the NP-labeled T cells around 1064 nm, and the absorbance increased with the AuNR concentration used for labeling (Figure 2D). We proceeded to assess PA imageability *in vitro* using tissue-mimicking phantoms, where the dome-shaped inclusions contained the cells. The PA spectrum for NP-labeled cells (OD = 10) from 680–970 nm (Figure 2E) agreed with the UV–vis results (Figure 2D). Qualitatively, US/PA overlay images at 1000 cells/μL for labeling at OD = 10 showed a stark PA signal increase compared to the mixed condition and unlabeled controls (Figure 3A). Quantitative analysis from the labeled T-cells (Figure 3B) indicated 2.1- and 10.1-fold increases in PA signal for the OD= 1 and OD = 5 labeling condition, respectively, relative to the case of OD = 10. Over a 30-fold increase in PA signal was observed for the OD = 10 labeling condition compared to the mixed and unlabeled conditions.

We then determined limits of detection at 1064 nm wavelength for each labeling condition in a serial dilution phantom of cell concentration (Figure 3C,D). A statistically significant increase in average PA signal compared to the unlabeled condition was observed at 125 and 250 cells/μL for OD = 10 and OD = 5 labeling conditions, respectively. The concentration of the initial T cell dose can easily exceed 100k cells/μL, corresponding to 1 × 10^7^ cells, in *in vivo* studies of ACT. Therefore, the *in vitro* results are encouraging, where limits of detection are 100-fold below the reported *in vivo* injection doses. At OD = 1, PA signals exceeded background, but the results were not statistically significant. We observed 3.3-fold and 2-fold increases for T cells labeled at OD = 1 compared to unlabeled controls for 1000 and 500 cells/μL. In summary, the NP-labeled T cells generate PA signals *in vitro*, where labeling conditions of OD = 10 and OD = 5 are favorable from an imaging perspective.

**Figure 4:**
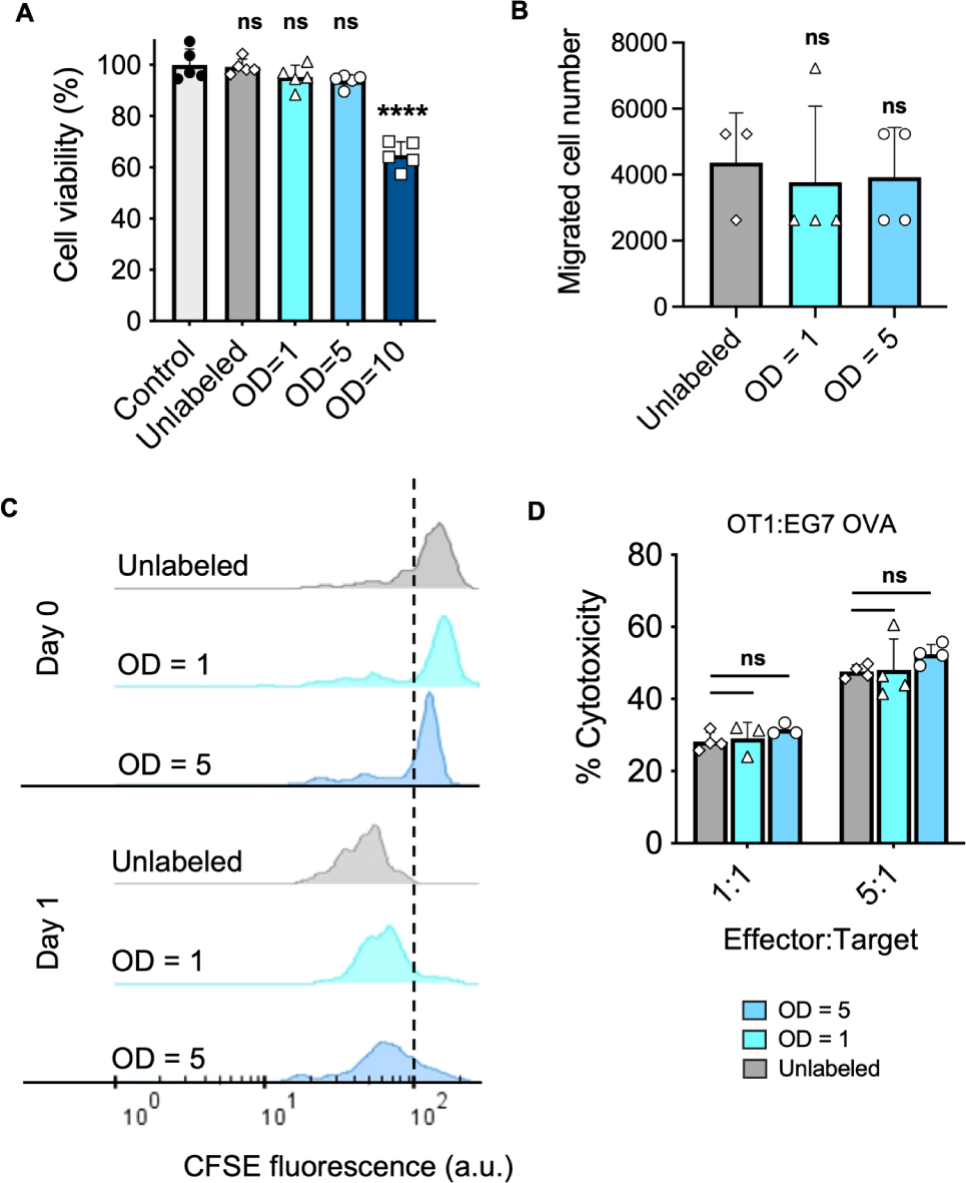
Gold nanorod labeling does not impact primary T cell function. (A) Cell viability immediately following labeling with mmAuNRs at OD = 1, 5, and 10. For labeling, T cells were coincubated with mmAuNRs at 30 min in serum-free RPMI medium at pH= 6.8. “Unlabeled” indicates T cells were incubated in the same slightly acidic RPMI used for labeling. “Control” indicates T cells remained in standard medium for 30 min. (B) T cell migration in a transwell. Unlabeled or NP-labeled T cells (OD = 1 or OD = 5) were seeded in the top transwell insert, and 50 ng/mL CXCL12 was added to the bottom well. After 24 h, T cells that migrated into the bottom chamber were counted. (C) T cell proliferation. T cells were stained with CFSE dye and labeled with mmAuNRs. Fluorescence was measured immediately postlabeling for a baseline reading (Day 0) and 24 h later (Day 1), where the peak shift in CFSE fluorescence represents dye dilution due to cell division. Similar proliferation trends were observed across conditions. (D) T cell killing assay to assess impact of NP labeling on T cell effector function. NP-labeled OT-1 T cells (OD = 1 and OD = 5) or unlabeled OT-1 T cells were coincubated with a cognate OVA-positive cancer cell line, EG7 OVA, at 5:1 and 1:1 Effector:Target (E:T) ratios, i.e., the ratio of T cells to cancer cells. In panels A, B, and D plotted values are the mean ± standard deviation. Data were analyzed by an unpaired Student’s *t* test comparing each group to its respective unlabeled control. * *p* < 0.05; ** *p* < 0.01; *** *p* < 0.001; ns = nonsignificant.

We next evaluated cytocompatibility to ensure NP-labeling does not impact T cell vitality or effector function. T cell viability (Figure 4A), migration (Figure 4B), proliferation (Figure 4C), killing of cancer cells (Figures 4D and S8), and cytokine secretion (Figures S9 and S10) were assessed. No impact on cell viability was observed compared to the control for labeling at OD ≤ 5. At OD = 10, T cell viability was reduced to 60% and remaining assessments were only conducted at OD = 5 and OD = 1. For the migration assay, primary murine T cells were labeled with AuNRs and added to the top chamber of a transwell, where the bottom chamber contained CXCL12 chemokine. After 24 h, no difference was observed in migration of labeled T cells to the bottom chamber for OD = 5 or OD = 1 compared to unlabeled controls (Figure 4B). NP labeling also did not impact cell proliferation, and similar cell doubling was observed via the CFSE assay over 24 h following NP labeling (Figure 4C). T cell killing assays were conducted with OT-1 primary murine T cells that specifically recognize and target OVA-positive cancer cell lines, i.e., EG7 OVA lymphoma (Figure 4D) or B16 OVA melanoma (Figure S8). Finally, cytokine secretion of interferon gamma (IFN-γ) and granzyme B was similarly assessed via coincubation of NP-labeled or unlabeled OT-1 T cells and B16 OVA melanoma cancer cells (Figures S9 and S10). Results indicate that NP-labeled T cells maintain effector functions. In summary, conjugation of silica-coated AuNRs to the T cell surface does not compromise key cellular functions under appropriate labeling conditions. Therefore, primary murine T cells were labeled with NPs at OD = 5 with a 30 min incubation time for *in vivo* studies to maximize cytocompatibility and imageability.

Prior to beginning *in vivo* US/PA imaging studies, we first validated our *in vivo* models using hand caliper measurements (Figure S11) to ensure expected trends in tumor growth and response to therapy were observed.^65^ NP-labeled OT-1 T cells were systemically delivered to mice bearing OVA-positive or OVA-negative flank tumors, i.e., EG7-OVA or EL4. In the antigen-positive case, the transferred T cells should recognize and target the tumor. Accordingly, the tumor volume decreased over time (Figure S11B). In the antigen-negative case, the transferred T cells do not match the tumor profile, and tumor volume increased over time (Figure S11B). In addition, we confirmed that NP labeling did not impact therapeutic response *in vivo* (Figure S11C), which corroborated *in vitro* cytocompatibility assays. After confirming expected trends in tumor growth and treatment response in both groups, we proceeded with *in vivo* US/PA imaging assessments.

**Figure 5:**
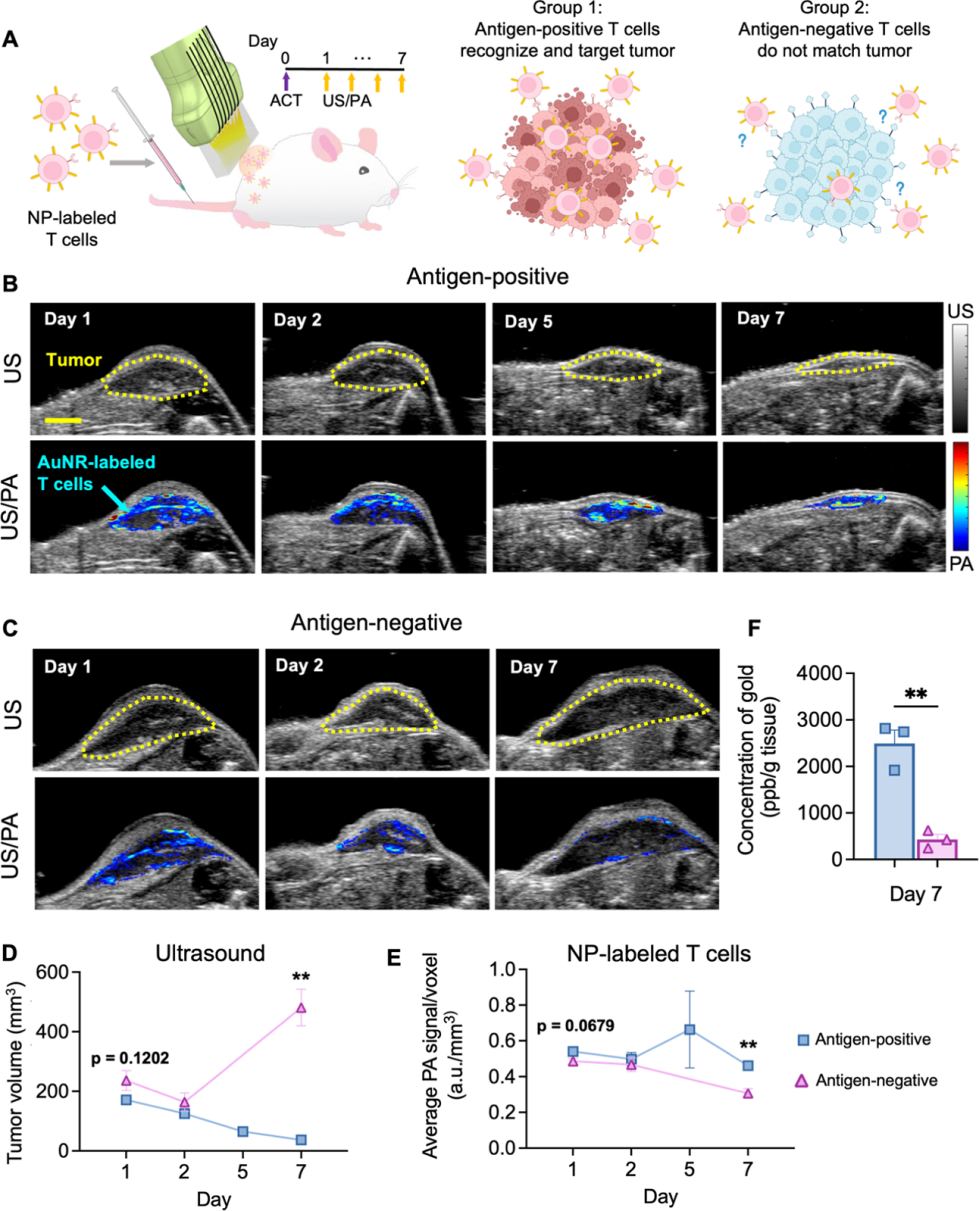
US/PA detects NP-labeled T cells at the primary tumor *in vivo* after ACT. (A) Schematic illustrating the workflow and set up of *in vivo* US/PA imaging experiments to monitor adoptively transferred NP-labeled T cells. (B, C) Ultrasound (grayscale, top row) and US/PA (color scale, 1064 nm wavelength, bottom row) overlay images, where PA signal represents accumulation of NP-labeled T cells within the primary tumor from Day 1 to Day 7. (B) Antigen-positive group, where mice were inoculated with EG7 OVA tumors and cognate OT-1 T cells labeled with NPs. (C) Antigen-negative group, where mice were inoculated with EL4 tumors, which are OVA-negative, therefore OT-1 T cells will not recognize and target EL4 tumors with the same efficiency. Note that US/PA images were not acquired at Day 5 for the antigen-negative cohort to give mice more recovery time to reach the Day 7 study end point. (D) Quantitative analysis of longitudinal changes in tumor volume determined by three-dimensional volumetric ultrasound data. (E) Quantitative analysis of longitudinal T cell accumulation based on three-dimension photoacoustic data at 1064 nm wavelength, which approximately corresponds to the peak optical absorption of the NP-labeled T cells. (F) ICPMS of the primary tumor harvested at Day 7 to evaluate accumulation of NP-labeled T cells. For the antigen-negative cohort, *n* = 3 mice. For the antigen-positive cohort, *n* = 4 mice. Plotted values represent the mean ± standard error of the mean. Data were analyzed by an unpaired Student’s *t* test comparing the antigen-positive group to the antigen-negative group at each time point. The P-value is displayed when 0.05≤ *p* ≤ 0.15. Otherwise, asterisks denote statistical significance, where * *p* < 0.05; ** *p* < 0.01; *** *p* < 0.001. Scale Bar = 3 mm. (A) was created in part with BioRender.

Our first goal was to assess accumulation of NP-labeled T cells at the primary tumor with PA imaging and accompanying changes in tumor volume with ultrasound. Upon reaching an average tumor volume of ∼200 mm^3^, NP-labeled T cells were systemically delivered. Volumetric US/PA data sets were acquired for 7 days postinjection. PA data at 1064 nm wavelength was used to distinguish NP-labeled T cells, according to the unique, peak optical absorption of the AuNRs. By Day 1, US/PA images visualized initial delivery of antigen-positive or antigen-negative, NP-labeled T cells at the tumor (Figures 5B,C and S12). However, quantitative analysis showed higher PA signal at the tumor in mice that received antigen-positive, NP-labeled T cells (*p* = 0.0679; Figure 5E), indicating initial differences in infiltration. By Day 7, qualitative differences in US/PA images were apparent. PA images showed that antigen-positive, NP-labeled T cells remained at the tumor, and a decrease in tumor size was observed in ultrasound (Figure 5B,D). On the contrary, minimal PA signal was observed at the tumor for the antigen-negative, NP-labeled T cells by Day 7, and ultrasound showed an increase in tumor size (Figure 5C,D). Quantitative analysis confirmed a statistically significant increase in PA signal by Day 7, indicating NP-labeled T cells were better retained for the antigen-positive group (Figure 5E). ICP-MS confirmed the presence and higher accumulation of antigen-positive, NP-labeled T cells at the tumor (Figure 5F). In summary, PA imaging enabled detection of NP-labeled T cells at the primary tumor *in vivo* following systemic delivery. Longitudinal monitoring of PA signal illustrates the ability to detect differences in retention of NP-labeled T cells, and changes in ultrasound tumor volume depict morphological differences in post-treatment response.

**Figure 6:**
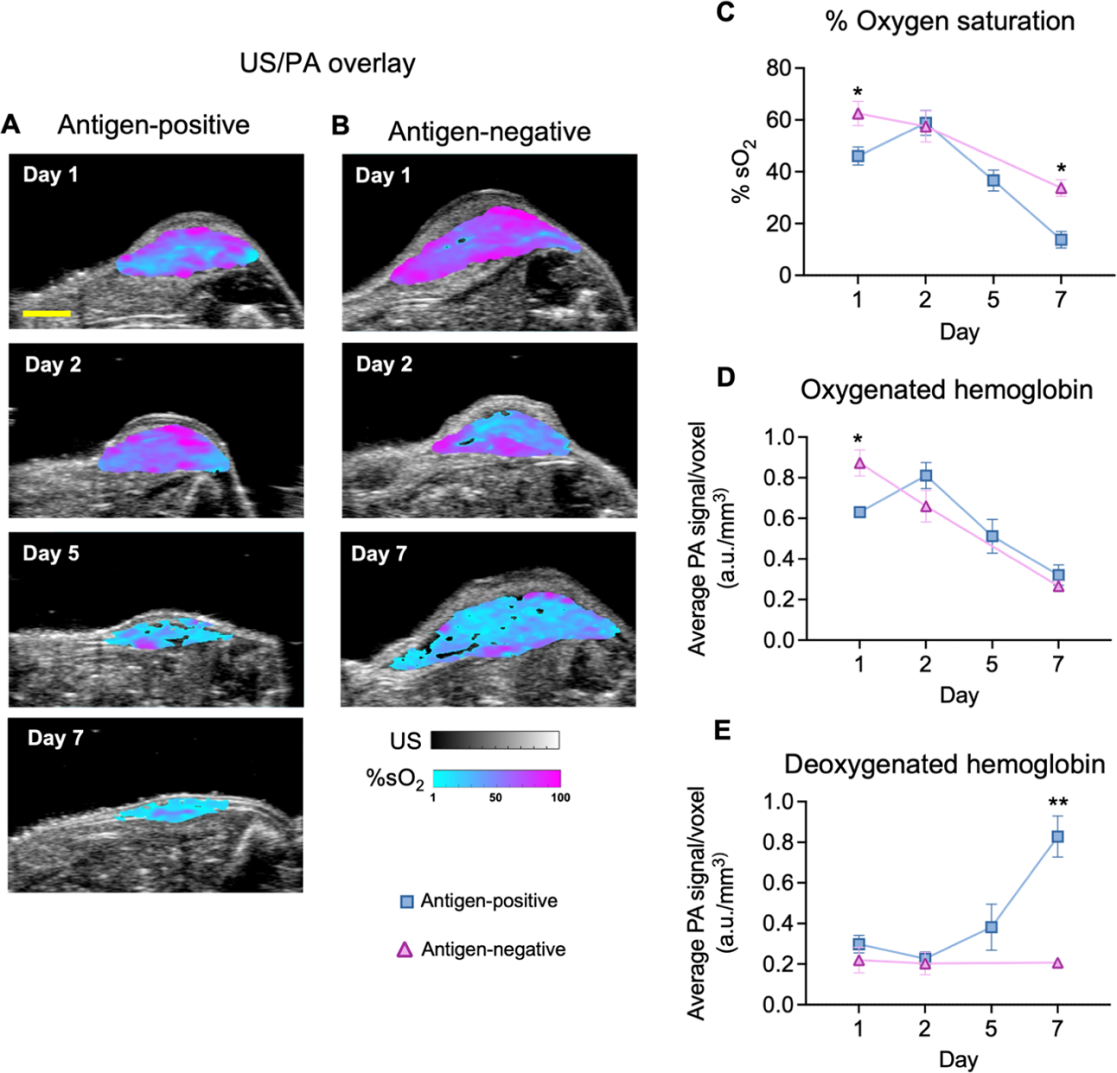
Differences are observed in US/PA blood biomarkers at the primary tumor *in vivo* after ACT of NP-labeled T cells. (A, B) Ultrasound (grayscale) and photoacoustic (color scale) overlay images indicating % oxygen saturation (%sO_2_) within the tumor to indicate region that are more normoxic (pink) or hypoxic (blue) following adoptive transfer of NP-labeled T cells. A) Antigen-positive group, where mice were inoculated with EG7 OVA tumors and cognate OT-1 T cells labeled with NPs. (B) Antigen-negative group, where mice were inoculated with EL4 tumors, which are OVA-negative, therefore OT-1 T cells will not recognize and target EL4 tumors with the same efficiency. Note that US/PA images were not acquired at Day 5 for the antigen-negative cohort to give mice more recovery time to reach the Day 7 study end point. Quantitative analysis of %sO_2_ (C), oxygenated hemoglobin (D), and deoxygenated hemoglobin (E) were determined via multiwavelength PA imaging and spectral unmixing to distinguish endogenous absorbers. Differences for each blood biomarker were observed based on the positive antitumor immune response stimulated by NP-labeled T cells in the antigen-positive group (blue lines) versus the antigen-negative group (pink lines). For the antigen-negative cohort, *n* = 3 mice. For the antigen-positive cohort, *n* = 4 mice. Plotted values represent the mean ± standard error of the mean. Data were analyzed by an unpaired Student’s *t* test comparing the antigen-positive group to the antigen-negative group at each time point. The P-value is displayed when 0.05≤ *p* ≤ 0.15. Otherwise, asterisks denote statistical significance, where * *p* < 0.05; ** *p* < 0.01; *** *p* < 0.001. Scale bar = 3 mm.

To expand our approach and evaluate post-treatment response beyond changes in ultrasound tumor volume, we next developed a multiplex imaging approach to simultaneously distinguish NP-labeled T cells and analyze accompanying changes in blood biomarkers (Figure 6). In this case, acquisition of multiwavelength PA data sets and spectral unmixing was required to distinguish oxygenated and deoxygenated hemoglobin. Single-wavelength imaging at 1064 nm (Figure 5) was still deployed to distinguish NP-labeled T cells. The ideal wavelengths and spectral unmixing parameters were determined *a priori* using intratumoral injections of NP-labeled T cells and unlabeled T cells (Figure S13). The former represents a positive imaging control. The latter represents a negative imaging control to ensure the unmixing algorithm minimizes crosstalk between endogenous, i.e., oxygenated and deoxygenated hemoglobin, and exogenous absorbers, i.e., AuNRs (Figure S13).

Multiwavelength three-dimensional US/PA data sets were acquired following ACT of NP-labeled T cells. Qualitative differences in % oxygen saturation were observed in mice that received antigen-positive (Figure 6A) or antigen-negative (Figure 6B) T cells. Excitingly, quantitative analysis highlighted statistically significant differences in PA signals corresponding to % oxygen saturation, oxygenated hemoglobin, and deoxygenated hemoglobin (Figure 6 C–E, respectively). Assessing changes in blood biomarkers (Figure 6) alongside changes in NP-labeled T cell infiltration and longitudinal retention (Figure 5) may further capture differences in response to treatment. When treatment was effective in the antigen-positive group, higher PA signal from NP-labeled T cells was accompanied by decreasing tumor volume, decreasing % oxygen saturation, decreasing oxygenated hemoglobin, and increasing deoxygenated hemoglobin over time. Alternatively, in the antigen-negative group, where treatment was ineffective, lower PA signal from the NP-labeled T cells was accompanied by the exact opposite trends–increasing tumor volume, increasing % oxygen saturation, increasing oxygenated hemoglobin, and decreasing deoxygenated hemoglobin. Although differences in ultrasound tumor volume, deoxygenated hemoglobin, and T cell infiltration were statistically different by Day 7, differences in % oxygen saturation and oxygenated hemoglobin were detected by Day 1 post cell transfer, which points to the potential to detect early response to therapies in future studies.

Here, we developed a US/PA imaging platform, augmented with nanomaterials, to enable *in vivo* noninvasive, longitudinal detection and monitoring of adoptive T cell therapies. We also highlight proof-of-concept for assessing post-treatment response with blood biomarkers. Our approach leverages silica-coated AuNRs, synthesized in-house, with enhanced photostability and optical properties for PA imaging to enable detection of T cells with high sensitivity.^46,47^ To allow deep tissue imaging, we synthesized AuNRs that absorbed in the second tissue optical window, NIR II, from 1000–1300 nm where scattering from biological tissues and endogenous absorption is low.^66−68^ Imaging depth and detection sensitivity can be further improved in this wavelength regime due to the higher allowable laser energies, up to 100 mJ/cm^2^ at 1064 nm wavelength, based on safety standards set by the American National Standards Institute (ANSI).^69^ However, tuning optical absorption to the NIR II window typically requires nanorods with a large size and high aspect ratio, where previous reports indicate ∼150 nm length by ∼25 nm width.^70−73^ Conventional AuNRs with these physical dimensions easily deform into nanospheres during pulsed laser irradiation, making longitudinal PA imaging unreliable due to spectral shifting.^46^ Here, our small AuNRs had dimensions of ∼50 nm length by ∼8 nm width with peak optical absorption at 1064 nm.^47^ Silica-coating further enhanced photostability. In addition, the small size of our AuNRs is important for minimizing interference with the immune synapse formation between T cells and cancer cells, where the nanoscale gap can exclude surface molecules. For this reason, smaller NPs are favorable, and a highly dense surface coating is not ideal.^74−76^

The AuNRs were further functionalized with maleimide groups to enable conjugation to the T cell surface (Figure 2). Although others have demonstrated T cell labeling by NP internalization via passive uptake or mechanical stimulation^77,78^ we gravitated toward a cell surface labeling approach to avoid the limited natural phagocytic activity of T cells. The surface labeling approach was inspired by methods previously developed by Stephan et al., where drug-loaded lipid nanoconstructs were conjugated to the T cell surface via thiol-maleimide linkages.^79^ We expanded on their approach to demonstrate safe, cell surface-conjugation of AuNRs to extend applications to PA imaging. Given thiol expression across a variety of cell types, the approach could be adapted to label other nonphagocytic cells.^79^ Labeling approaches were validated in Jurkat T cells and primary murine T cells. Maleimide–thiol conjugation was critical to enable NP-T cell labeling. When T cells were incubated with silica-coated AuNRs without maleimide functionalization, no detectable PA signal was observed, comparable to unlabeled controls (Figure 3B). Our results support that the NPs are primarily conjugated to the T cell surface. However, given their small size, some NPs could be internalized.^78^ In any case, PA imageability should not be impacted if cells are labeled by NP internalization or surface conjugation. Our group has previously demonstrated success in various applications of cell tracking with internalized NPs.^50−53,56,57,59,61^ Finally, T cell vitality and effector function *in vitro* (Figures 4 and S8–S10) and *in vivo* (Figure S11C) was not impacted by NP labeling.

Finally, *in vivo* imaging studies highlighted the ability to detect the NP-labeled T cells with longitudinal US/PA imaging *in vivo* following systemic delivery. It is known that information on lymphocyte location, infiltration, and status at the primary tumor site is predictive of clinical outcomes.^29,80−82^ A combination of single-wavelength PA imaging at 1064 nm and multiwavelength PA imaging allowed us to detect NP-labeled T cells and analyze changes in endogenous absorption from blood biomarkers, including % oxygen saturation, deoxygenated hemoglobin, and oxygenated hemoglobin. Ultrasound quantified corresponding changes in tumor volume and provided critical anatomical context to visualize infiltration and retention of NP-labeled T cells. To develop and validate our approach, our US/PA imaging platform was tested in different tumor models, where different responses to therapy were expected.^65^ The antigen-positive group represented a scenario of response to therapy. Mice bearing an OVA-positive tumor received T cells with the cognate receptor to enable recognition and targeting. In this case, US/PA results depicted a decrease in tumor volume, % oxygen saturation, and oxygenated hemoglobin, and an increase in deoxygenated hemoglobin over time. On the contrary, the antigen-negative group represented a scenario of tumor evasion. Mice bearing an OVA-negative tumor received T cells with a mismatched receptor, and the exact opposite trends were found across all US/PA imaging biomarkers. Excitingly, results demonstrate that our US/PA imaging platform can detect T cell delivery and provide information on therapy progression.

Previous work from Kim et al. used similar tumor models in their ACT studies to track Cy5.5 tagged T cells using NIR fluorescence imaging.^65^ Their results similarly depicted differences in accumulation and retention of Cy5.5 tagged T cells in therapy responders and nonresponders. Trends in delivery and retention of Cy5.5 tagged T cells agreed with our results with NP-labeled T cells. Like the aforementioned study, we similarly defined therapy response based on a decrease or increase in tumor volume. In the future, we aim to expand our US/PA approach to understand and stratify nuanced responses to therapy in complex, heterogeneous tumor models, which is an important step to inform downstream treatment planning. Toward this goal, the combination of single-wavelength imaging and multiwavelength spectral sweeps highlights a key advantage of PA imaging.

In any cell tracking application, imaging signal dilution due to cell proliferation is a concern. As labeled T cells divide, the PA imaging signal will drop, which inherently limits the capabilities of longitudinal monitoring. However, according to prior studies on T cell kinetics following ACT^65,83,84^ maximal T cell infiltration at the primary tumor occurs within the first 72 h post-transfer. Our current results (Figures S4 and 5) support that NP-labeled T cells can be detected at these critical early time points. However, further development of the imaging platform will more deeply investigate capabilities for long-term imaging to extend the monitoring window.

Optical contrast of PA imaging opens up a variety of opportunities to implement multiplex imaging approaches by leveraging unique optical spectra of exogenous and endogenous absorbers.^30^ Multiplex capabilities are a defining feature of PA imaging compared to existing modalities in the immunoimaging field, i.e., MRI, CT, PET. Beyond cell tracking and assessment of blood biomarkers, the platform can be expanded by designing additional exogenous contrast agents with distinct spectral features to simultaneously probe the molecular profile of the tumor environment or create more sophisticated constructs for assessing cell status.^85−90^

Our study validates the feasibility of our US/PA imaging approach to track adoptively transferred T cells and highlights differences in US/PA biomarkers to provide insights on post-treatment response. Later, methods can be expanded toward earlier identification of responders or nonresponders to therapy to improve guidance of adoptive T cell therapy, inform downstream treatment planning, and increase the likelihood of a positive therapeutic outcome. Toward development of advance theranostic imaging platforms, multifunctional nanoconstructs could be designed and leveraged to enhance T cell potency, prevent exhaustion, or modulate the tumor microenvironment under image-guidance. Together, our US/PA imaging approach to monitor nanoparticle-engineered T cells in the context of cancer treatment represents an exciting, versatile platform with potential to guide and enhance adoptive cellular immunotherapies across a variety of applications.

## Conclusion

We have developed a US/PA imaging platform, augmented with nanomaterials, to enable *in vivo* noninvasive, longitudinal monitoring of adoptive T cell therapies. The platform leverages silica-coated AuNRs with favorable properties for PA imaging to enable sensitive detection of NP-labeled T cells deep within the primary tumor. A cell surface engineering approach was developed to conjugate the AuNRs to the T cell surface via maleimide–thiol linkage. T cells were safely labeled with the AuNRs without impacting vitality or effector functions *in vitro* or *in vivo*. The NP-labeled T cells were systemically administered in mice bearing different tumor types to demonstrate the feasibility of our US/PA imaging approach to monitor adoptively transferred T cells and assess post-treatment response. Our *in vivo* imaging approach deploys single-wavelength PA imaging to distinguish the NP-labeled T cells and multiwavelength PA imaging to evaluate the corresponding changes in blood biomarkers for multiplex imaging. Ultrasound provides critical anatomical context to visualize the NP-labeled T cell accumulation and changes in the tumor volume. NP-labeled T cell infiltration was successfully detected with our US/PA imaging approach, alongside longitudinal changes in tumor volume, % oxygen saturation, and deoxy-/oxygenated hemoglobin. Furthermore, different trends were observed across these imaging biomarkers in mice that received antigen-positive or antigen-negative T cells. Taken together, the results point to the exciting potential of the US/PA imaging approach to identify early response to therapy or create advanced theranostic platforms.

## Methods and Experimental

### Materials

Gold chloride trihydrate (HAuCl_4_, Sigma-Aldrich), sodium citrate tribasic dihydrate (Sigma-Aldrich), cetyltrimethylammonium bromide (CTAB, VWR), hydrochloric acid (HCl, Sigma-Aldrich), nitric acid (HNO_3_, GFS Chemicals), silver nitrate (AgNO_3_, Sigma-Aldrich), sodium borohydride (NaBH_4_, Sigma-Aldrich), hydroquinone (Sigma-Aldrich), tetraethyl orthosilicate (TEOS, Sigma-Aldrich), sodium hydroxide (NaOH, Sigma-Aldrich), methanol (Sigma-Aldrich), toluene (Sigma-Aldrich), silane-PEG-maleimide 2 kDa (MW 2000 Creative PEGWorks), polyethylene glycol thiol (mPEG-SH, MW:2000, Biochempeg), Dulbecco’s Modified Eagle Medium (DMEM, Cytvia), Roswell Park Memorial Institute 1640 medium (RPMI 1640, Cytvia), bovine serum albumin (BSA, Sigma-Aldrich), Cell Tracker Green CMFDA (Thermo Fisher Scientific), 3-(4,5-dimethylth-iazol-2-yl)-2,5-diphenyltetrazolium bromide (MTT, EMD Millipore), lactate dehydrogenase assay kit (LDH, Abcam), fetal bovine serum (FBS, Sigma-Aldrich), penicillin/streptomycin (Sigma-Aldrich), red blood cell (RBC) lysis buffer (BioLegend), nonessential amino acids (NEAA, Thermo Fisher Scientific), sodium pyruvate (Thermo Fisher Scientific), 2-mercaptoethanol (Sigma-Aldrich), recombinant murine SDF-1α (CXCL12, PeproTech), recombinant human interleukin-2 (IL-2; > 1 × 10^7^ units/mg, PeproTech; or 1 × 10^6^ units, Frederick National Laboratory), ovalbumin (OVA 257–264, chicken, Sigma-Aldrich), phosphate buffered saline (PBS, Sigma-Aldrich), gelatin (MP Biomedicals), and silica (0.25 μm diameter, Sigma-Aldrich) were used in accordance with manufacturer’s instructions.

### Ultrasound and Photoacoustic (US/PA) Imaging System

All combined US/PA images were acquired using a Vevo LAZR system (Fujifilm VisualSonics, Inc.) at a frame rate of 5 frames per second using a linear array ultrasound transducer (center frequency = 20 or 30 MHz; LZ250 or LZ400, respectively) with integrated optical fibers to enable PA imaging. The laser source was a Q-switched, Nd:YAG-pumped optical parametric oscillator (OPO) with a laser pulse repetition frequency of 20 Hz and a laser pulse duration of 7 ns. Single-wavelength US/PA images were acquired at 1064 nm wavelength. Multiwavelength spectral sweeps were acquired from 680–970 nm wavelengths in 5 nm increments for two-dimensional scans.

To generate 3D US/PA data sets, the transducer was connected to a translational motor, and two-dimensional US/PA scans were acquired every 0.121 mm over the entire volume of interest. The two-dimensional scans were then compiled to form 3D volumetric data sets. Due to file size restrictions for acquisition of 3D US/PA data sets, multiwavelength, volumetric data sets were acquired from 700–900 nm wavelengths in 25 nm increments.

### Synthesis of Maleimide-Modified Silica-Coated Gold Nanorods

AuNRs were synthesized according to previously reported methods.^46,47,91^ All glassware and stir bars were cleaned with aqua regia (3:1 HCl to HNO_3_) and fully dried prior to use. All solutions were prepared in deionized ultrafiltered water (DIUF), unless otherwise noted. Briefly, CTAB solution (0.1M) was prepared by heating for 30 min above 35 °C. Once fully solubilized, the solution was cooled to room temperature. Under magnetic stirring, 10 mL CTAB, 0.2 mL HAuCl_4_ (0.02 M), and 25 μL AgNO_3_ (0.1 M) were added to the reaction vessel, and the reagents were stirred for 5 min. Next, 7 μL of HCl (1 M) were added followed by 525 μL of hydroquinone (0.1 M), at which point the solution changes from yellow to colorless. After 15 min, 0.4 μL of ice cold NaBH_4_ was added to the reaction (0.05 M) and stirred for 3 min. Then, the reaction was left to incubate at 30 °C water bath for at least 16 h until peak optical absorption at 1064 nm was observed. The CTAB-stabilized AuNRs were washed twice with DW via centrifugation for 20 min at 18,500 rcf to reduce the final CTAB concentration to below 0.001 M in a volume of 10 mL.

AuNRs were silica-coated with a mesoporous layer, referred to as AuNR@mSiO_2_, via a modified Stöber method.^46,62,63,92−94^ From the previous step, 5 mL of washed AuNRs were added to a small, clean glass vial. Under magnetic stirring at room temperature, 10 μL of NaOH (1 M) were added to the AuNR solution, followed by 20 μL of 20% TEOS solution in anhydrous methanol. The reaction was capped tightly and stirred. After 20 min, an additional 20 μL of 20% TEOS solution in anhydrous methanol was added. The reaction was capped tightly and stirred for another 20 min, at which point, 20 μL of 20% TEOS solution in anhydrous methanol was added. After the addition of the third and final allotment of TEOS, the reaction proceeded overnight. The following day, the reaction was quenched, and the nanoparticles were washed in methanol via centrifugation for 20 min at 10,000 rcf three times to remove residual CTAB. After the final washing step, residual methanol was evaporated to obtain the final AuNR@mSiO_2_.

Silica-coated gold nanorods (AuNR@mSiO_2_), were functionalized with maleimide immediately prior to cell labeling experiments according to previously reported method, with slight modifications.^95^ AuNR@mSiO_2_ were resuspended in toluene (1 mg/mL) in a round-bottom flask and heated to 110 °C under reflux. Silane-PEG-maleimide (0.1 mg per ml AuNR@mSiO_2_) and silane-mPEG (0.9 mg per ml AuNR@mSiO_2_) were added and stirred for 24 h. The next day, the maleimide-modified NPs were resuspended and diluted in an equal volume of methanol, and then transferred to a 1.5 mL microcentrifuge tube. The sample was washed in methanol via centrifugation at 10,000 rcf for 20 min, repeated 4 times. After the final wash, the particles were resuspended in sterile PBS at the desired optical density for cell labeling.

### Nanoparticle Characterization

Nanoparticle size and morphology was characterized using transmission electron microscopy (TEM, HT7700, Hitachi). TEM images were acquired using the Gatan Digital Micrograph software. TEM samples were prepared using drop casting, where 5 μL of the nanoparticle solution in methanol was dropped on a copper mesh grid, followed by air-drying overnight. Nanoparticle surface functionalization was assessed via zeta-potential measurements using dynamic light scattering (DLS, Zetasizer Nano ZS, Malvern Instruments Ltd.). Optical properties, specifically the optical absorption spectrum and measurements of optical density, were determined via UV–vis-NIR spectrophotometry (Evo 220, Thermo Fisher Scientific).

### Nanoparticle Photostability Assessments

To assess photostability, nanoparticle samples were added to a 96-well plate with black sides. The samples were separated by at least one empty well on each side to avoid unintentional laser (Vibrant, Opotek) irradiation of nearby samples. Samples were irradiated at 1064 nm optical wavelength, corresponding to the peak optical absorbance of the NPs. Each individual sample was carefully aligned and positioned directly below the optical fiber. The distance between the sample and optical fiber was constant. The laser beam coming out of the optical fiber was collimated such that the spot size corresponded to the diameter of the well. Laser fluence was controlled by adjusting the flashlamp Q-switch delay timing and adding neutral density filters (ThorLabs, Inc.), as needed. Fluence was verified using an external energy meter (Nova II, Ophir). The laser was fired for 20 pulses, 100 pulses, or 500 pulses to irradiate separate samples at various fluences. Then, the absorbance spectrum was measured via UV–vis-NIR spectrophotometry (Evo 220, Thermo Fisher Scientific).

### Culture of Immortalized Cell Lines

Cells were incubated under standard conditions at 37 °C in a 5% CO_2_ humidified incubator (Heracell VIOS 160I, Fisher Scientific). In all cases, culture media was changed every 2–3 days. Adherent cell lines (B16 OVA and B16 F10) were passaged at ∼80–90% confluency. To passage, cells were detached by incubating with 0.05% Trypsin-EDTA (Corning) for 5 min. Trypsin was neutralized by quenching with complete medium followed by washing via centrifugation at 125*g* for 5 min. Cells were subcultured at a 1:10 ratio. Suspension cell lines (Jurkats) were maintained at 1 × 10^5^ – 1 × 10^6^ cells/ml.

Jurkats, EG7 OVA, and EL4 (ATCC TIB39) cell lines were cultured in RPMI-1640 medium supplemented with 10% fetal bovine serum and 1% penicillin/streptomycin. EL4 cells (ATCC TIB39) were cultured in DMEM supplemented with 10% fetal bovine serum and 1% penicillin/streptomycin. B16 F10 and B16 OVA cell lines were cultured in DMEM supplemented with 10% fetal bovine serum and 1% penicillin/streptomycin.

### Isolation and Expansion of Primary Murine OT-1 T Cells

Primary murine T cells were isolated and expanded following previously established protocols.^96,97^ Splenocytes were isolated from OT-1 transgenic mice (Strain #003831 Jackson Laboratory). Briefly, mice were euthanized and the spleen was harvested. In the biosafety cabinet, the isolated spleen was mashed and chopped into fine, millimeter sized chunks before pipetting through a 40 μM cell strainer into a 50 mL centrifuge tube. The filtered cell suspension was centrifuged at 1000 RCF for 5 min and resuspended in 5 mL of 1x RBC lysis buffer to deplete red blood cells. The remaining splenocytes were pelleted and filtered again through the 40 μM cell strainer. The splenocytes were counted and suspended at a final concentration of 2 × 10^6^ cells/ml in complete T cell media supplemented with 100 units/mL IL-2 and 1 μg/mL OVA peptide. After 2 days, T cells were maintained at 1 × 10^6^ cells/mL in media supplemented with 100 units/mL IL-2. T cells were ready for use in experiments on Day 6 postisolation. The complete T cell medium consists of RPMI-1640 with glutamine supplemented with 10% FBS, 1% penicillin/streptomycin, 1% NEAA, 1% sodium pyruvate, and 50 μM 2-mercaptoethanol.

### T Cell Labeling with Nanoparticles

Primary murine OT-1 T cells were isolated and expanded as described above. On Day 6 post isolation, OT-1 T cells were labeled with NPs. Jurkats were labeled following the same labeling approach below. Cells were suspended at a concentration of 10 × 10^6^ cells/ml in serum-free RPMI 1640 supplemented with 1% penicillin/streptomycin at pH adjusted to 6–6.5. An equal volume of maleimide-functionalized NPs was added to the cells at the desired optical density, i.e., NPs at a stock concentration of OD = 20, 10, or 2 were added to achieve a OD = 10, 5, or 1, respectively. The cell solution was incubated at 37 °C and 5% CO_2_ for 30 min. Every 10 min, cells were briefly pipetted to resuspend. The labeling reaction was quenched by adding an equal volume of mPEG-thiol (MW 2K at 10 mg/mL) solution in PBS and incubating for 10 min in a humidified CO_2_ incuabtor. The cells were then washed twice for 5 min at 1000 RCF. Following the final wash, cells were resuspended in the appropriate solution, i.e., media or sterile PBS, at the desired concentration for downstream studies.

Optical properties of labeled cells were evaluated my measuring the extinction spectrum using UV–vis-spectrophotometry (Evo 220, Thermo Fisher Scientific) and comparing to the extinction spectrum of unlabeled cells. Cell surface labeling was analyzed via SEM (Hitachi SU8010). To prepare SEM samples, labeled and unlabeled cells were first fixed in 4% paraformaldehyde solution for 10 min. The cells were washed via centrifugation to remove excess formalin. Then, the fixed cell samples were progressively dehydrated through brief incubation with 10%, 30%, 50%, 70%, 90%, 100% ethanol. The dehydrated cell samples were redispersed in 1 mL of ethanol. The cell samples were dropcast onto silicon wafers overnight (Electron Microscoy Sciences) and sputter-coated with a gold–platinum layer of 7 nm thickness prior to acquiring SEM images. The concentration of gold on the cell surface was analyzed via ICP-MS (Agilent Technologies). Postlabeling, cell samples were suspended at 1000 cells/μL in 1 mL of aqua regia and incubated overnight to dissolve the nanoparticles. The concentrated aqua regia solution was then diluted 20-fold in DW and filtered using a 0.45 μm PTFE syringe filter prior to ICP-MS analysis to remove any remaining cellular debris.

Cell surface labeling was further analyzed using an endocytosis inhibitor. Prior to nanoparticle labeling, primary OT-1 T cells were treated with dynasore (Millipore Sigma) for 20 min (100 μM) at room temperature, according to a previously published approach.^98^ Following dynasore treatment, T cells were then incubated with the maleimide-modified gold nanorods (OD = 5). After 30 min, cells were washed via centrifugation to remove residual nanoparticles from the media. A tissue-mimicking gelatin dome phantom was prepared to assess PA signals.

### Evaluation of Cytocompatibility Following Nanoparticle Labeling of T Cells

Immediately postlabeling, T cell vitality and function were assessed via standard assays for viability, migration, proliferation, killing of cancer cells, and cytokine secretion. In all cases, the cells were washed via centrifugation to remove any residual nanoparticles.

For viability of T cells, the MTT assay was deployed according to the manufacturer’s rapid protocol (EMD Millipore) for suspension cells. Briefly, cell samples (*n*= 5 samples per labeling condition) were added to a 96-well plate and pelleted. The media was aspirated and exchanged for MTT-containing media (0.5 mg/mL in 100 μL total volume) followed by incubation for 6–8 h. The media was then thoroughly mixed with 100 μL of DMSO, and allowed to stand for 30 min before taking the absorbance reading. The absorbance at 590 nm was measured using a multiwell plate reader (Synergy HT, BioTek).

A transwell assay was used to assess migration according to previously reported methods.^96,99^ In a 24-well plate, 600 μL of completed media containing CXCL12 chemokine at 50 ng/mL supplemented with 100 units/ml IL-2 was added to each well, referred to as the lower chamber. A transwell insert with 6.5 mm diameter and 5 μM pore size (Corning) was placed at the top of each well, referred to as the upper chamber. T cell samples were added to the upper chamber at 1000 cells/μL in 100 μL complete media supplemented with 100 units/ml IL-2. The next day, roughly 18 h later, the number of cells in the lower chamber, representing the number of T cells that migrated from the upper chamber, were counted (*n* = 3 counts per condition) using the Countess 3 (Thermo Fisher Scientific).

The CellTrace CFSE Cell Proliferation Kit (Thermo Fisher Scientific) was used to assess cell proliferation. CFSE was protected from light throughout the procedure. Primary murine T cells were stained with CFSE according to the manufacturer’s instructions, where cells at a concentration of 1 × 10^6^ cells/ml in PBS were incubated with CFSE (5 μM) for 20 min at 37 °C and 5% CO_2_. The reaction was quenched by adding serum-containing medium at 5x the original volume of the PBS staining solution and incubating for 5 min. The cells were pelleted and resuspended in complete medium supplemented with 100 units/mL IL-2. Cells were counted using the Countess 3 Automated Cell Counter (Thermo Fisher Scientific). Fluorescent measurements were acquired using the green light cube. Baseline brightness settings for fluorescence were established immediately after CFSE staining and NP-labeling of the T cells. These baseline readings served as the Day 0 fluorescent measurements, and the same settings were used for readings acquired 24 h later. The fluorescent data was gated based on the brightfield images to only include cells above 7 μM diameter in the fluorescent counts. The data was exported as a.fcs file for analysis in FlowJo version 10 (BD Biosciences). Data was displayed as a historgram to depict shifts in the peak fluorescence, where cell proliferation and subsequent generations are expected to have a peak at lower fluorescent concentrations.

T cell effector function was assessed using a killing assay. Target cancer cells were seeded in at 96-well plate at 20,000 cells per well. Adherent target cells, i.e., B16 OVA or B16 F10, were seeded the day prior to coincubation. Suspension cells, i.e., EG7 OVA or EL4, were seeded immediately prior to the addition of T cells. Labeled T cells or unlabeled controls were then added to the target cells at ratios of 5:1 or 1:1. After 24 h of coincubation, the MTT assay (B16 OVA and B16 F10) or LDH assay (EG7 OVA and EL4, Abcam) were used to assess % cytotoxicity of the cancer cells according to the manufacturer’s instructions.

Interferon gamma expression was assessed in labeled and nanoparticle-labeled OT-1 cells via an ELISA. B16 F10 and B16 OVA cancer cells were plated at a cell density of 100,000 cells in a 12-well plate. To investigate the effect of nanoparticle labeling on interferon gamma expression, unlabeled or nanoparticle-labeled OT-1 T cells were coincubated with B16 F10 or B16 OVA cancer cells for 24 h at an effector to target ratio of 5:1. To quantify secretory interferon gamma from OT-1 T cells, the coculture supernatant of labeled or nanoparticle-labeled T cells with B16 F10 or B16 OVA cancer cells was collected, followed by an ELISA analysis with a mouse interferon gamma ELISA kit (Invitrogen) according to the manufacturer’s protocol.

Granzyme B expression in cancer cells was assessed via flow cytometry analysis following coincubation with OT-1 T cells. B16 F10 and B16 OVA cancer cells were prestained with CFSE according to the manufacturer’s protocol (Thermo Fisher Scientific) and plated at a cell density of 100,000 cells in a 12-well plate. To investigate the effect of nanoparticle labeling on granzyme B activity against target cancer cells, unlabeled or nanoparticle-labeled primary T cells isolated from OT-1 donor mice were coincubated with B16 F10 or B16 OVA cancer cells for 24 h at an effector to target ratio of 5:1, followed by intracellular staining using the intracellular fixation and permeabilization buffer (BioLegend) and APC-tagged granzyme B antibody (BioLegend) according to the manufacturer’s protocol. The granzyme B activity in B16 F10 and B16-OVA cancer cells was characterized by flow cytometry (Cytek Aurora).

### Preparation of Tissue-Mimicking Phantoms for US/PA Imaging

To evaluate US/PA signal from NPs or NP-labeled cell samples *in vitro*, a tissue mimicking gelatin dome phantom was created based on previously reported methods.^100^ Briefly, a phantom base composed of 8% gelatin (MP Biomedicals) and 0.2% silica (Sigma-Aldrich) by mass was prepared by heating solutions in DIUF H_2_O above 40 °C while stirring until the gelatin fully dissolved. The base layer was poured into a plastic container, bubbles were removed, and the base was refrigerated to solidify. Inclusions were prepared by making a 16% gelatin solution and mixing with an equal volume of sample, i.e., NPs or labeled cells, to achieve a final gelatin concentration of 8%. The inclusion samples were kept in a liquid state by warming at 40 °C. Once the base layer was solid 20–40 μL volumes of the inclusion samples were pipetted on top of the base to form a dome that contained the sample of interest. The phantom, now consisting of the base and sample inclusions, was solidified again via refrigeration. At least 3 domes were prepared per sample.

For imaging, the phantom container was filled DIUF H_2_O. The US/PA transducer was submerged in the water for coupling and any bubbles were removed from the transducer face. The transducer height was positioned to ∼9 mm above the inclusions, and the height was held constant throughout the imaging session to maintain constant fluence. Multiwavelength US/PA data sets were acquired from 680–970 at 5 nm intervals, and single-wavelength US/PA data sets were acquired at 1064 nm wavelength. All data was processed using VevoLAB 5.7.0 software or exported to MATLAB version R2021b (MathWorks) for postprocessing.

### ACT Experiments

All animal procedures were approved by the Institutional Animal Care and Use Committee (IACUC) at the Georgia Institute of Technology in accordance with federal guidelines for the care and use of laboratory animals. Female C57BL/6J (Jackson Laboratory) mice at 6–8 weeks old were inoculated with 1 × 10^6^ EG7 OVA or EL4 cancer cells in 100 μL of sterile saline via subcutaneous injection in the left flank. Mice were observed every 2–3 days and tumors were measured by hand using calipers. The tumor volume was calculated by the modified ellipsoidal equation, where V = 0.5 x length x width^2^. Upon reaching an average volume of 200 mm^3^, all mice were sublethally lymphodepleted prior to adoptive cell transfer by total body irradiation at 100 cGy/min for 3 min and 51 s (RAD Source RS2000; 160 kV).^96^ OT-1 T cells were labeled with NPs at OD = 5, and the NP-labeled cells were prepared immediately prior to transfer. NP-labeled T cells were suspended in 100 μL of sterile saline and were systemically administered via the tail vein. The concentration of NP-labeled T cells was adjusted between 1000–5000 cells/μL to achieve the target total does of 1 × 10^6^ or 5 × 10^6^ T cells. Control groups received saline injections or unlabeled T cells following the same procedure. Over the next 72 h following ACT, all mice received 6 doses of 100,000 units of IL-2 (Peprotech) in 100 μL injection volume administered intraperitoneally in the morning and evening.

At Day 7 post adoptive cell transfer, mice were euthanized and tumors were harvested. To prepare samples for ICP-MS, according to previously reported methods,^101^ the excised tumors were weighed and then fixed in neutral buffered formalin for at least 24 h. Tumor tissue was rinsed and washed with PBS to remove excess formalin. Then, tumors were placed in 10 mL aqua regia and incubated using MARS Xpress 5 digestion microwave system (CEM Corporation) until the tissue was fully dissolved. The solution was diluted 20-fold with DW and filtered through a 0.45 μm PTFE syringe filter to remove any residual tissue. The concentration of Au ions, representing the AuNRs and reflecting accumulation of NP-labeled T cells, was analyzed by ICP-MS (Agilent) per gram tissue.

### *In Vivo* US/PA Imaging and Data Analysis

US/PA images were acquired at Day 0, 1, 2, 5, and 7 post transfer. For the antigen-negative group (mice inoculated with EL4 tumors and treated with OT-1 T cells), the Day 5 time point was eliminated because mice were extremely slow to recover from anesthesia after Day 2 imaging. Although humane end points were not reached, the increasing tumor burden and multiple days of consecutive anesthesia were impacting animal health. Thus, Day 5 images were not acquired to best ensure mouse survival up to the Day 7 study end point for the antigen-negative group.

Mice were lightly anesthetized with isoflurane to immobilize for imaging and secured to a small animal heating pad. The tumor was covered with ultrasound gel to couple to the US/PA imaging transducer. Three-dimensional volumetric scans were acquired at 1064 nm for single wavelength imaging or from 700–900 nm in 25 nm increments for multiwavelength scans. A two-dimensional multiwavelength scan was also acquired from 680–970 nm wavelengths in 5 nm increments to assist with development and optimization of spectral unmixing algorithms. After each imaging session, mice were allowed to recover before returning to the housing facility. All data sets were exported to MATLAB R2021b (MathWorks) for postprocessing. To focus the image analysis on the primary tumor, PA signals within the tumor volume were segmented using the MATLAB Volume Segmenter Toolbox, based on anatomical information from 3D ultrasound data sets. This segmentation delineates the tumor volume for both single-wavelength and multiwavelength data sets.

For multiwavelength three-dimensional scans, the segmented tumor PA data was filtered with an averaging filter with dimensions 6 pixels x 11 pixels x 3 frames or 225 μm × 495 μm × 360 μm, where the filter size corresponds to roughly three times the axial and lateral resolution of the imaging transducer. The data was then median filtered to remove any large noise spikes. The kernel size for the median filter was 3 pixels x 3 pixels or 111 μm × 135 μm. Data was thresholded to remove PA signal below the noise floor. The noise floor was defined as PA _min_ + 0.02(PA _max_ - PA _min_). Following segmenting, filtering, and thresholding, the multiwavelength data was then unmixed using linear least regression. Solutions were restricted to positive values. The optical absorption spectrum used in the linear fit were obtained from the Oregon Medical Laser Center (omlc.org) for oxygenated hemoglobin and deoxygenated hemoglobin.^102^ The PA spectrum of NP-labeled T cells used in the linear fit was from multiwavelength PA images of NP-labeled T cells suspended in Matrigel acquired after subcutaneous injection *in vivo*.

### Statistical Analysis

All statistical analyses were performed using Microsoft Excel, R, or GraphPad Prism version 10.1.1 (GraphPad Software). Details of the specific statistical test corresponding to each data set and the number of replicates are included in the corresponding figure caption. In all cases, confidence interval limits are indicated with asterisks, where * *p* < 0.05, ** *p* < 0.01, *** *p* < 0.001, and n.s. = not significant.

## Data Availability

Data that support the findings of this study will be made available from the corresponding author upon reasonable request.

## Supporting Information Available

Further assessments of AuNR photostability and PA signal enhancement following silica-coating; Characterization of PA imageability of NP-labeled Jurkat T cells *in vitro*; Additional studies of cytocompatibility following NP-labeling of Jurkat T cells, including viability, migration, and cell division; SEM images of NP-labeled primary murine T cells; Further assessments to verify T cell surface labeling via treatment with an endocytosis inhibitor; Additional killing assay with NP-labeled T cells and melanoma cells; ELISA of IFN-γ cytokine release in NP-labeled T cells; Flow cytometry to assess granzyme B expression in cancer cells following coincubation with NP-labeled T cells; Caliper measurements of tumor volumes to validate the mouse model system used for *in vivo* US/PA imaging studies; Verification that PA signal from NP-labeled T cells is above background and accompanying imaging controls to develop spectral unmixing algorithms (PDF)

## Abbreviations

ACT: adoptive cell therapy
ANSI: American National Standards Institute
Au: gold
AuNR: gold nanorod
CAR: chimeric antigen receptor
CT: computed tomography
CFSE: carboxyfluorescein succinimidyl ester
DIUF: deionized ultrafiltered, DIUF
GzmB: granzyme B
IACUC: Institutional Animal Care and Use Committee
ICP-MS: inductively coupled plasma mass spectrometry
IFN-γ: interferon gamma
MRI: magnetic resonance imaging
NIR: near-infrared
NP: nanoparticle
OD: optical density
PA: photoacoustic
PET: positron emission tomography
SPECT: single photon emission computed tomography
SEM: scanning electron microscopy
TEM: transmission electron microscopy
US: ultrasound
