# Supplementary material for: In Vivo Ultrasound and Photoacoustic Imaging of Nanoparticle-Engineered T Cells and Post-Treatment Assessment to Guide Adoptive Cell Immunotherapy
Source: ACS Nano. 2025 Feb 5;19(6):6079–94. doi: 10.1021/acsnano.4c12929 (PMC11841050; doi:10.1021/acsnano.4c12929)
Supplement: Supplementary file 1 — nn4c12929_si_001.pdf [file nn4c12929_si_001.pdf]

Supporting Information

In vivo ultrasound and photoacoustic imaging of nanoparticle-engineered T cells and post-treatment assessment to guide adoptive cell immunotherapy.

Kelsey P. Kubelick,[1,2,†] Jinhwan Kim,[1,2,†] Myeongsoo Kim,[1] Xinyue Huang,[1] Chenxiao Wang,[3] Seoyoon Song,[1] Younan Xia,[1,3,4] and Stanislav Y. Emelianov[1,2,*]

[1] *Wallace H. Coulter Department of Biomedical Engineering, Georgia Institute of Technology and Emory University School of Medicine, Atlanta, GA 30332, USA*

[2] *School of Electrical & Computer Engineering, Georgia Institute of Technology, Atlanta, GA 30332, USA*

[3] *School of Chemistry and Biochemistry, Georgia Institute of Technology, Atlanta, GA 30332, USA*

[4] *School of Chemical and Biomolecular Engineering, Georgia Institute of Technology, Atlanta, GA 30332, USA*

[†] These authors contributed equally to this work

[*] Corresponding Authors: Stanislav Y. Emelianov

✉E-mail: stas@gatech.edu

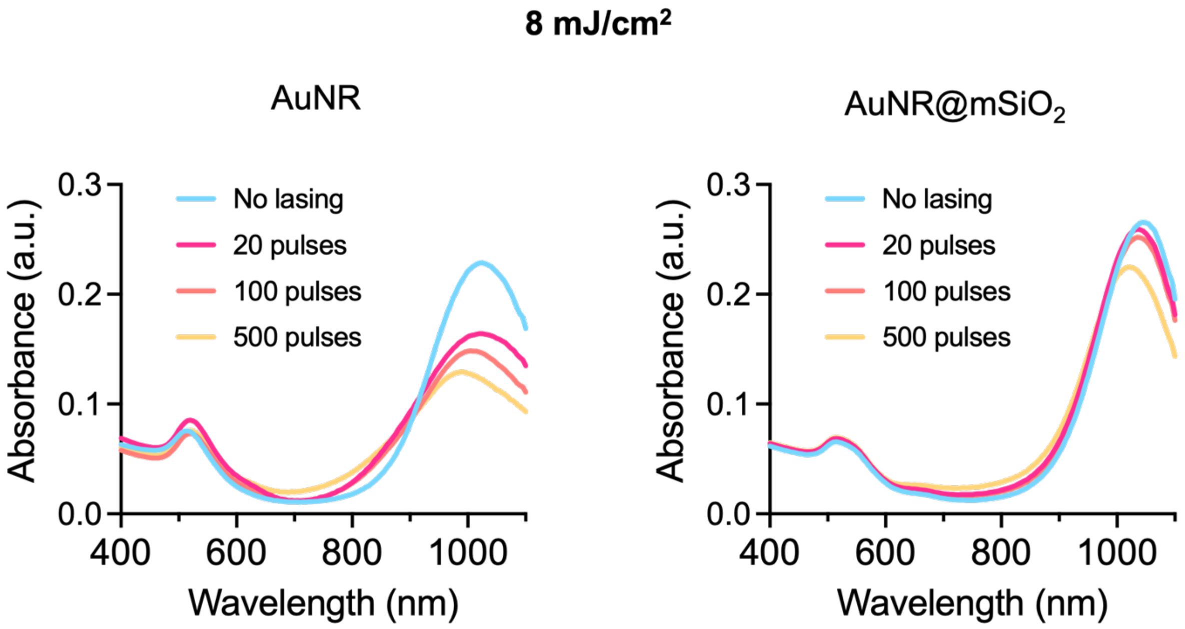

Figure S1. Silica-coating improves stability of AuNRs under pulsed laser light irradiation. UV-Vis spectrophotometry measurements were taken after irradiating bare AuNRs (left panel) or silica-coated AuNRs (AuNR@mSiO_2; right panel) with 20, 100, or 500 laser pulses at a fluence of 8 mJ/cm^2. Compared to the no lasing control (blue line), a decay in the peak optical absorption was observed for the bare AuNRs after only 20 pulses (pink line). The optical absorption of the silica-coated AuNRs remained stable up to 500 laser pulses (yellow line).

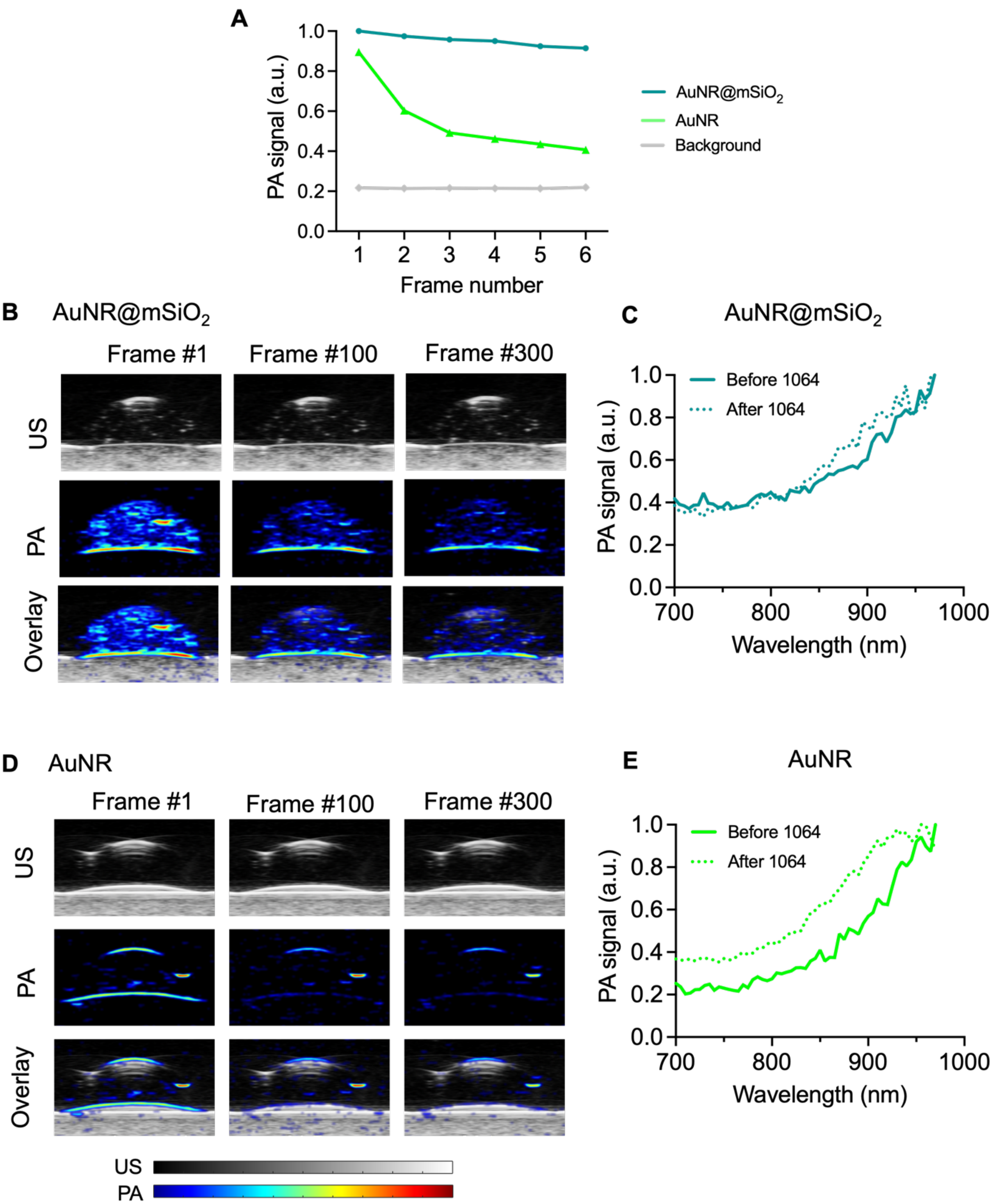

Figure S2. PA analysis indicates silica-coating improves stability of AuNRs during imaging. A tissue mimicking-phantom was prepared and inclusions (40 µl volume and ~5 mm wide) contained silica-coated AuNRs (AuNR@mSiO_2) or bare AuNRs at the same optical density. Both types of

nanorods were continuously irradiated by pulsed laser light at 1064 nm, corresponding to the peak optical absorption, λ_{max}, of the AuNRs. (A) After only 6 pulses, the PA signal from bare AuNRs (light green line) decays by nearly 50%, but minimal decay in PA signal was observed for the silica-coated AuNRs (dark green line). (B) Representative US (top row), PA (middle row), and US/PA overlay (bottom row) images of the gelatin dome inclusions containing silica-coated AuNRs. (C) PA spectrum of silica-coated AuNRs before (blue curve) and after (red curve) 1064 nm pulsed laser irradiation. A minimal shift in the PA spectrum was observed, indicating stability of the silica-coated AuNRs. (D) Representative US (top row), PA (middle row), and US/PA overlay (bottom row) images of the gelatin dome inclusions containing bare AuNRs. (E) PA spectrum of bare AuNRs before (blue curve) and after (red curve) 1064 nm pulsed laser irradiation indicates destabilization of the AuNRS, indicated by the shift in spectral signature.

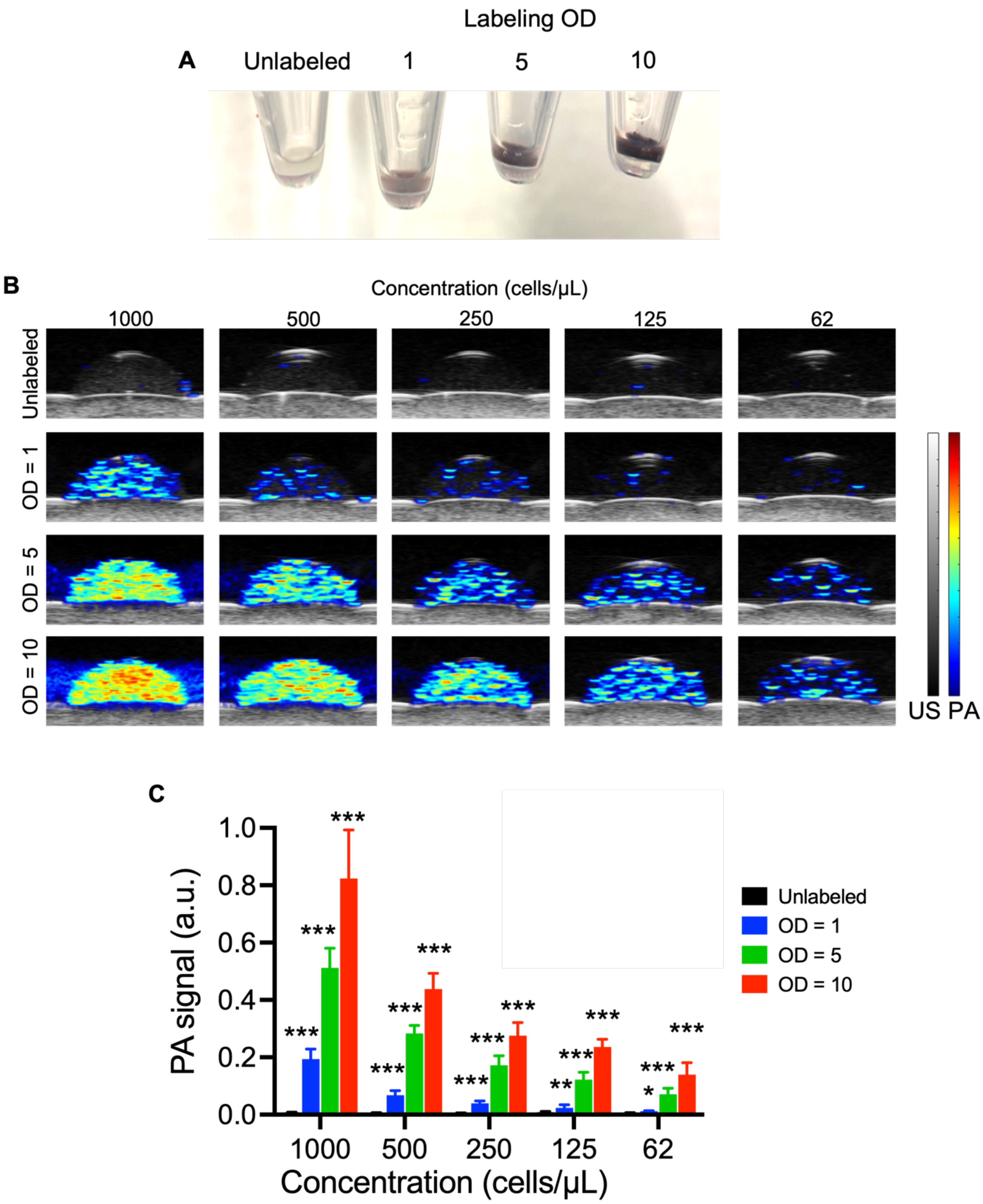

Figure S3. mmAuNRs interact with Jurkat T cells for successful labeling and PA imaging *in vitro*. (A) Photographs of the cell pellet of Jurkat T cells that were unlabeled, or incubated with maleimide-modified, silica-coated AuNRs (mmAuNRs) at optical density (OD) of 1, 5, or 10 (left

to right). A darker cell pellet was observed for cells incubated with higher concentrations (OD) of the mmAuNRs, indicating successful nanoparticle cell labeling. A tissue mimicking-phantom was prepared and inclusions (40 µl volume and ~5 mm wide) contained unlabeled Jurkat T cells (top row) or Jurkat T cells labeled with mmAuNRs at OD = 1, 5, or 10. All US/PA data was acquired at 1064 nm optical wavelength. (B) Representative US/PA overlay images of the cell dilution phantom to assess imaging sensitivity, where inclusions contained different concentrations of Jurkat T cells subject to different labeling conditions. (C) Corresponding quantitative analysis of (B) for each condition. A statistically significant increase compared to unlabeled controls was observed for all labeling densities (n = 3) down to 62 cells/µl. Plotted values are mean ± standard deviation. Data were analyzed by an unpaired student's T test comparing each group to its respective unlabeled control at each concentration. * $p<0.05$; ** $p<0.01$; *** $p<0.001$; ns = non-significant.

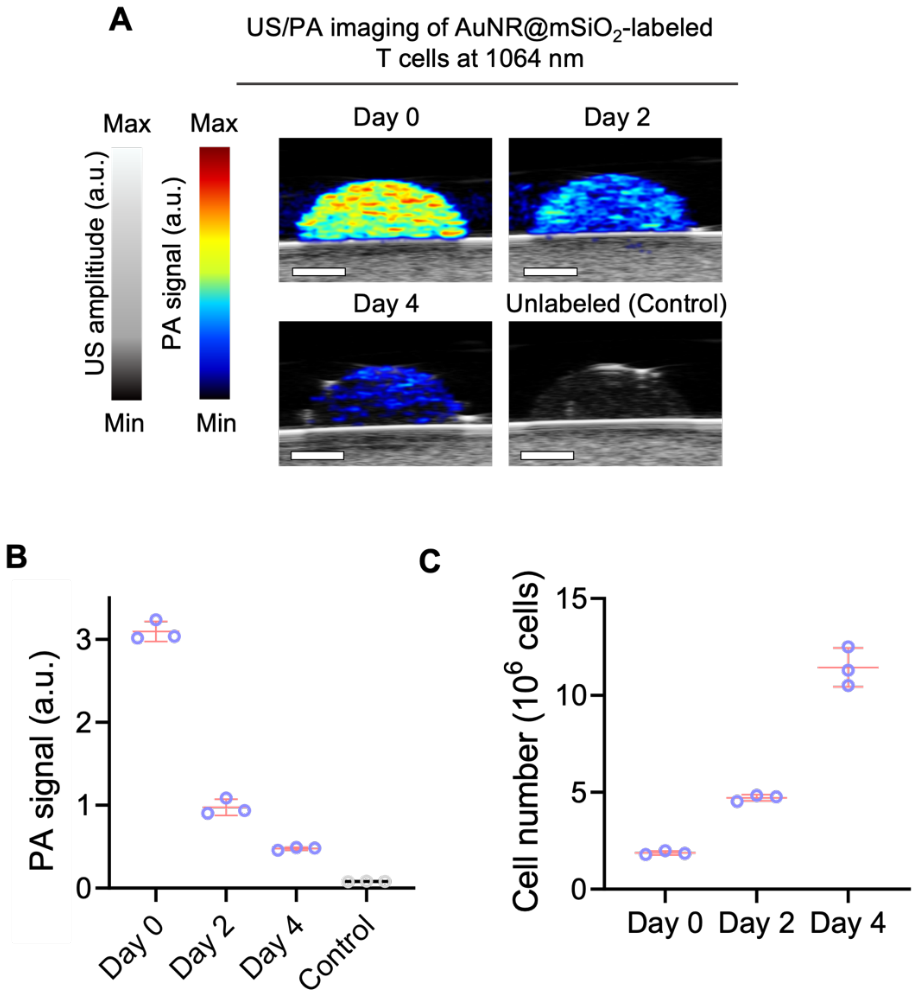

Figure S4. PA signal is maintained as NP-labeled T cells double. Jurkat T cells were labeled with mmAuNRs at an optical density of 5 and maintained in culture. Each day, a portion of the labeled T cells were siphoned from the culture and fixed. Cell samples from each time point were prepared in dome-shaped inclusions (40 µl volume at 1000 cells/µl; n = 3). A) Combined US/PA overlay images of NP-labeled T cells at Day 0, Day 2, and Day 4 compared to the unlabeled control. B) Quantification verifies that PA signal from NP-labeled T cells remains above background. C) Trends in PA signal dilution align with the doubling of the NP-labeled T cells, verified by cell counts. Scale bar = 2 mm.

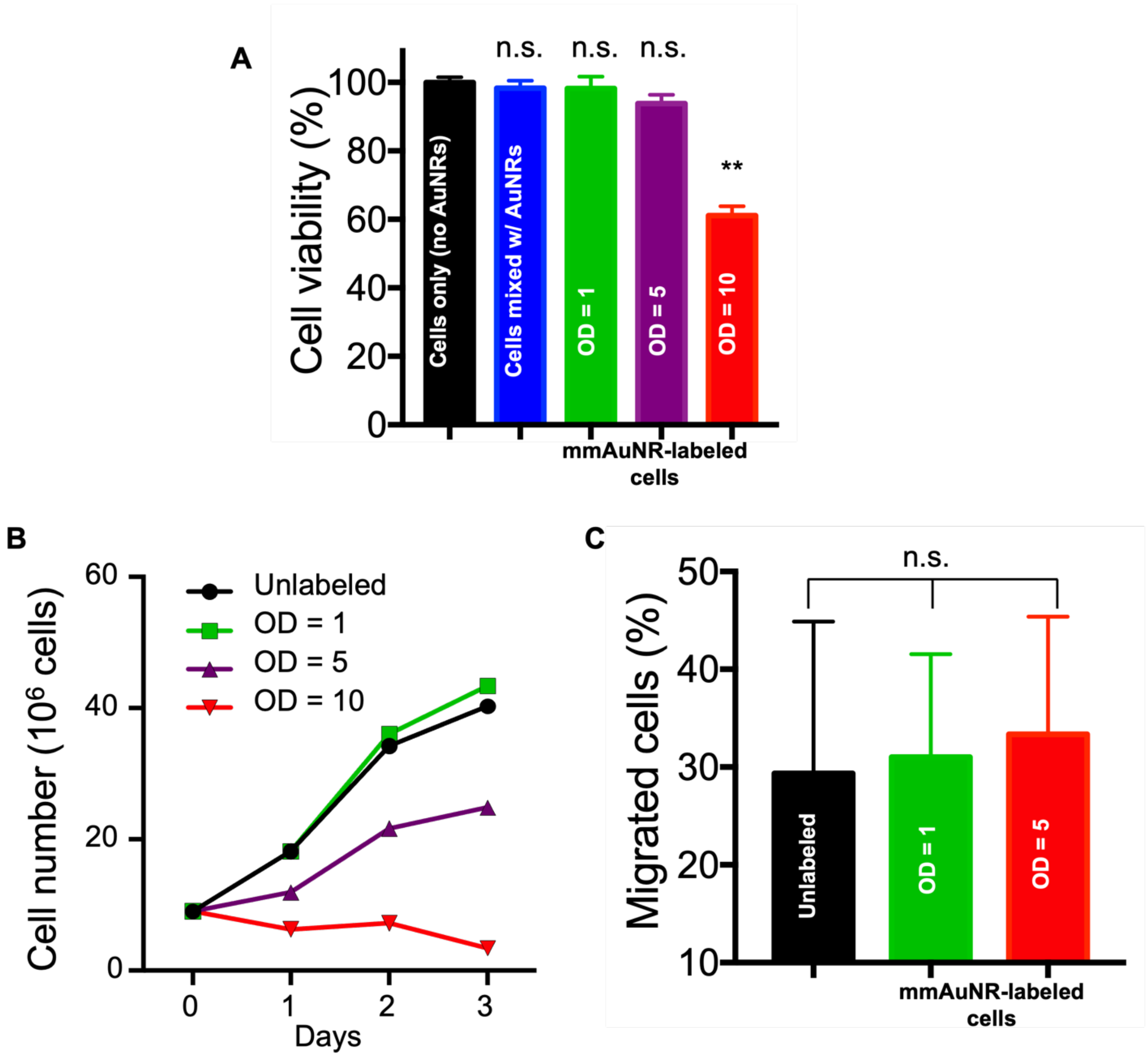

Figure S5. mmAuNR labeling of Jurkat T cells does not impact cell function with appropriate parameters. (A) MTT assay of cell viability of Jurkat T cells immediately following labeling with mmAuNRs at OD = 1, OD = 5, and OD = 10. The mixed condition indicates Jurkat T cells were incubated with non-functionalized nanorods at OD = 10. The labeling time was 30-minutes. Compared to the cell only (unlabeled) control (black bar), viability was not impacted by mmAuNR labeling up until an OD = 10. (B) For the proliferation assay, cells were labeled and counted on Day 0. The labeled cells were counted on each consecutive day. Similar proliferation trends were

observed for the unlabeled condition (black curve) and the OD = 1 condition (green curve). (C) Transwell migration assay, where Jurkat labeled T cells were seeded in the top well, and 50 ng/ml CXCL12 was added to the bottom well. After 24 hours, cells in the bottom well, representing the migrated cells, were counted. Data were analyzed by an unpaired Student's T test comparing each group to the cells only group in panel (A) or the unlabeled cells in panel (C). n = 5 for each condition. * $p<0.05$; ** $p<0.01$; *** $p<0.001$; ns = non-significant.

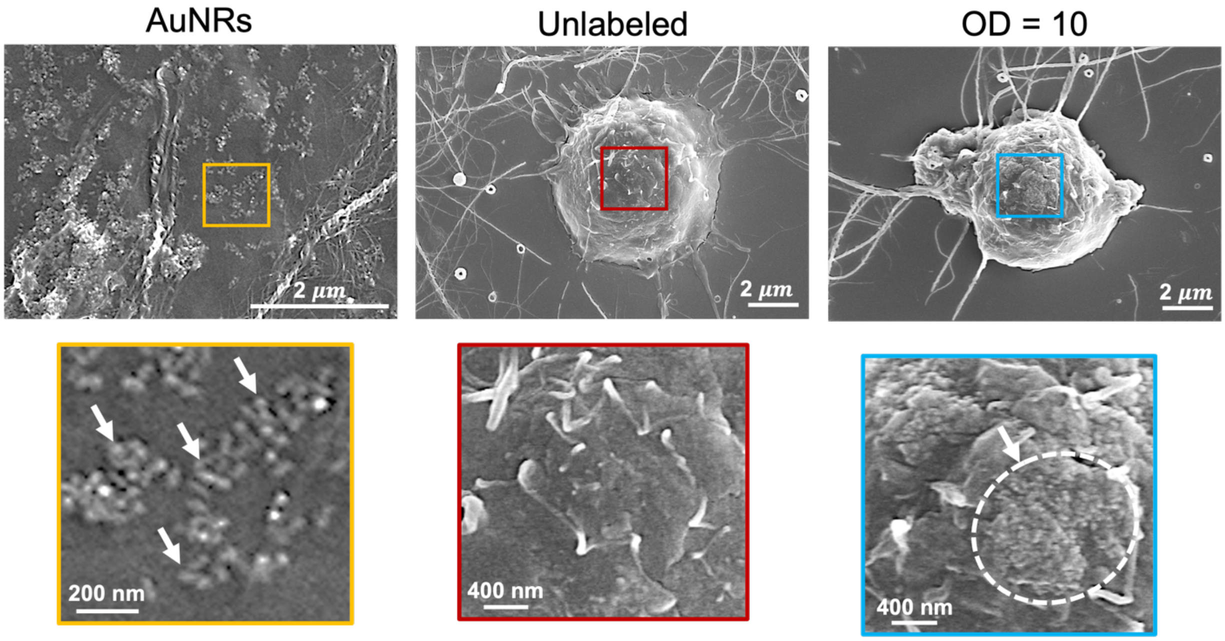

Figure S6. Visualizing mmAuNR labeling of primary murine T cells with SEM. Left to right - SEM images of AuNRs, unlabeled T cells, and NP-labeled T cells (OD = 10). Zoomed in regions of interest are denoted by the colored boxes. AuNRs are denoted by white arrows or circular regions of interest (white dashed line). Rice-shaped particles are visible on the surface of labeled T cells, giving a "fuzzy" appearance.

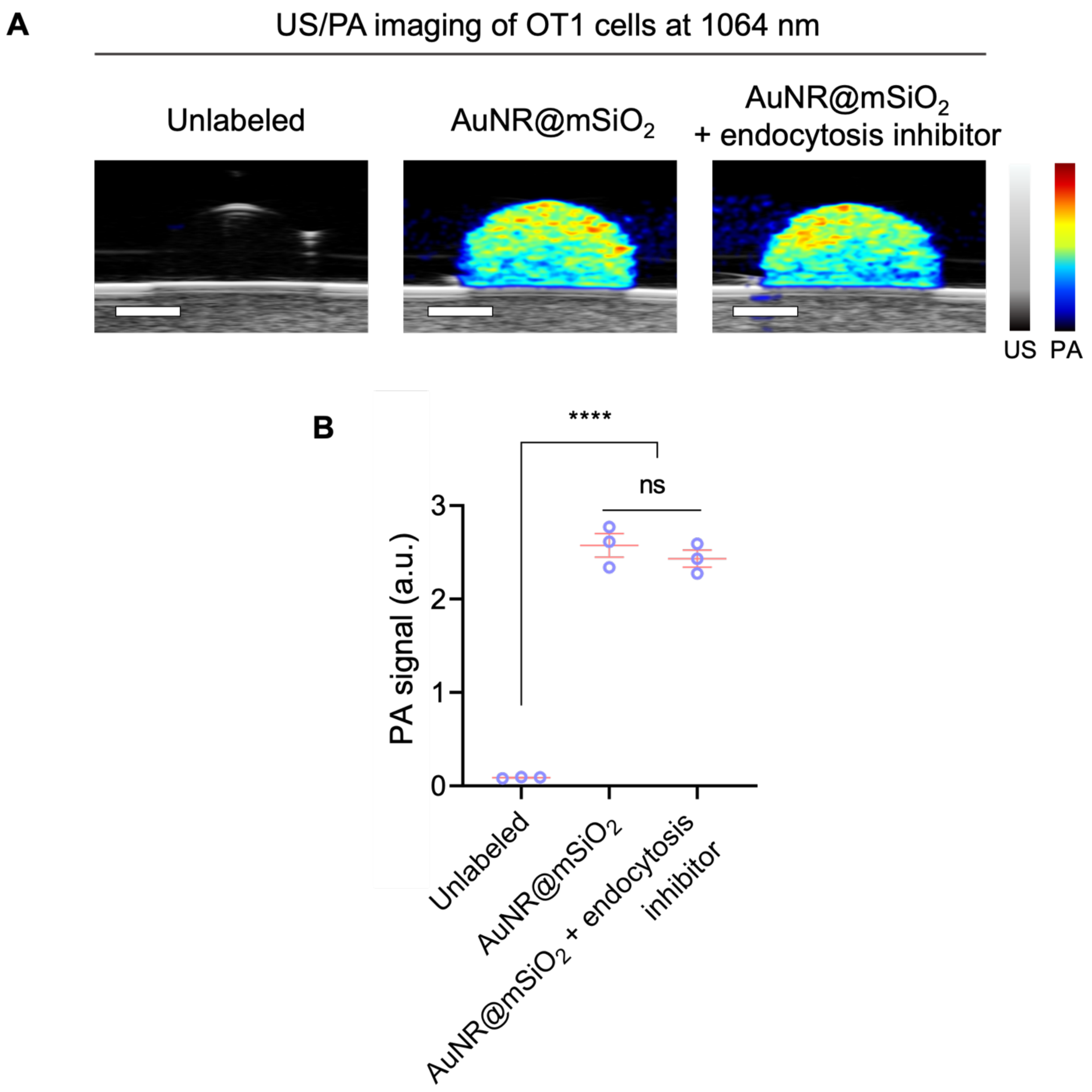

Figure S7. T cells that were pre-treated with an endocytosis inhibitor can still be labeled with nanoparticles to generate PA signals. Primary murine OT-1 T cells were treated with dynasore, an endocytosis inhibitor, prior to incubation with maleimide-modified AuNR@mSiO$_2$ nanoparticles. T cells were co-incubated with nanoparticles at OD = 5 for 30 minutes and washed to remove residual nanoparticles. Samples (n = 3 per condition) were prepared in 40 µl gelatin

domes at 1k cells/μl for US/PA imaging. A) US/PA overlay images of unlabeled T cells (left), untreated T cells co-incubated with AuNR@mSiO_2 (middle), and T cells treated with the endocytosis inhibitor and co-incubated with AuNR@mSiO_2 (right). High PA signals were observed following T cell co-incubation with nanoparticles in samples that were untreated or treated with the endocytosis inhibitor. B) Quantification of PA signals. The difference between T cells incubated with AuNR@mSiO_2 and T cells pre-treated with endocytosis inhibitor and incubated with AuNR@mSiO_2 was not significant. Results indicate that T cell labeling is independent of endocytosis, supporting a surface labeling mechanism. Scale bar = 2 mm. Plotted values are the mean ± standard deviation. The statistical analysis was conducted using a one-way ANOVA with Tukey post-hoc test. * $p<0.05$; ** $p<0.01$; *** $p<0.001$; ns = non-significant.

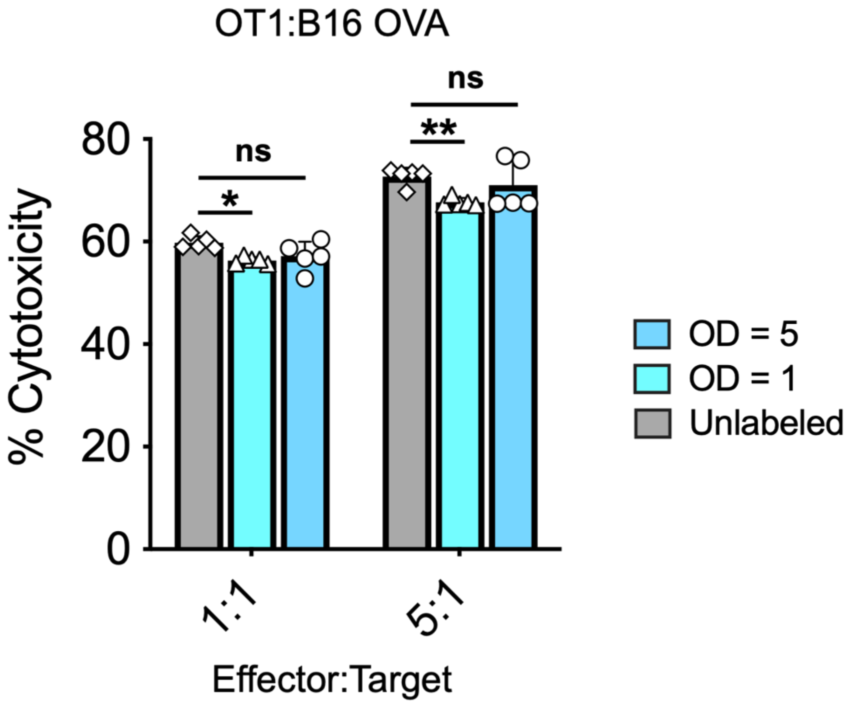

Figure S8. T cell killing assay in a melanoma cancer line to assess impact of NP labeling on T cell effector function. NP-labeled OT-1 T cells (OD = 1 and OD = 5) or unlabeled OT-1 T cells were co-incubated with a cognate OVA-positive cancer cell line, B16 OVA at 5:1 and 1:1 Effector:Target (E:T) ratios, i.e. the ratio of T cells to cancer cells. Plotted values are the mean ± standard deviation. Data were analyzed by an unpaired Student's T test comparing each group to its respective unlabeled control. * $p<0.05$; ** $p<0.01$; *** $p<0.001$; ns = non-significant.

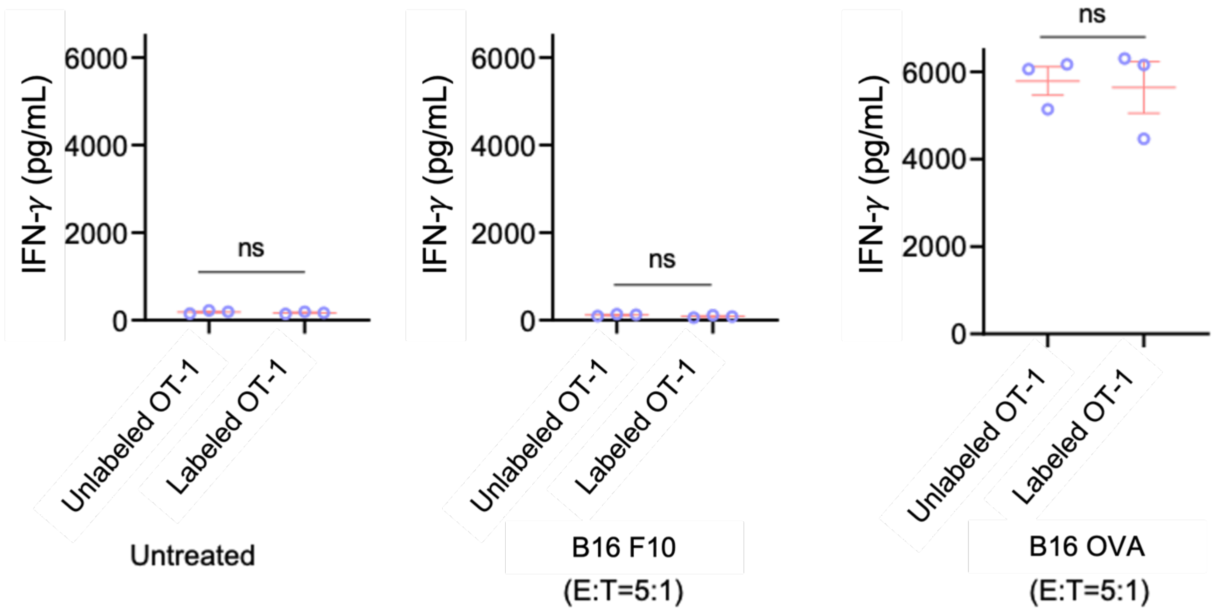

Figure S9. ELISA of IFN-γ cytokine release in NP-labeled T cells. As expected, minimal cytokine release was observed for unlabeled or labeled T cells alone, where "untreated" indicates no co-incubation with cancer cells (left panel). NP-labeled and unlabeled T cells were co-incubated with antigen-negative B16 F10 cancer cells (middle panel) or antigen-positive B16 OVA cancer cells (right panel). No significant difference was observed in IFN-γ secretion in unlabeled vs. labeled controls. NP-labeling has negligible impact on cytokine secretion. Data were analyzed by an unpaired Student's T test comparing each group to its respective unlabeled control. ns = non-significant.

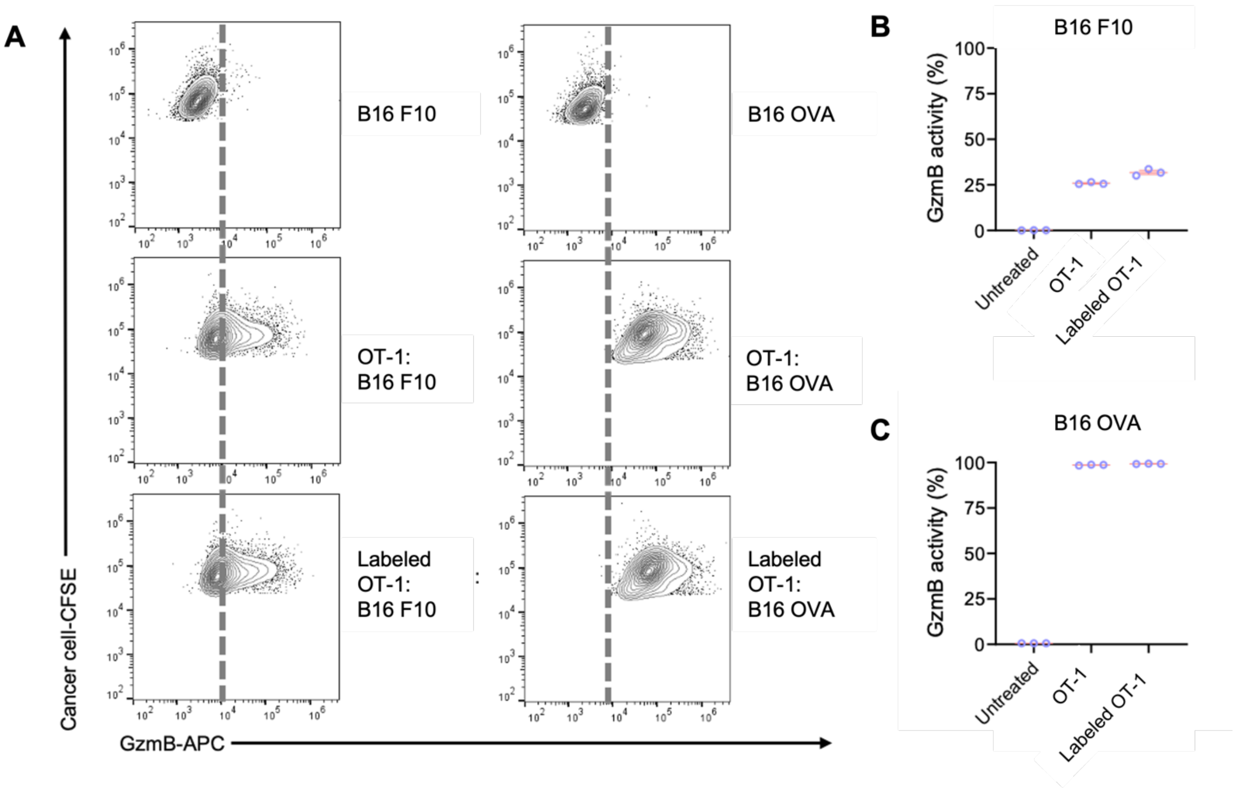

Figure S10. Flow cytometry to assess granzyme B (GzmB) expression in cancer cells following co-incubation with NP-labeled T cells. T cells were co-incubated with cancer cells at a ratio of 5:1. In all panels, "OT-1" indicates unlabeled T cells, and "labeled-OT-1" indicates nanoparticle-labeled T cells. A) As expected, when unlabeled T cells were co-incubated with antigen-positive B16 OVA cancer cells, an increase in GzmB expression was observed compared to cancer cells alone. Similar GzmB expression was observed in B16 OVA cancer cells following co-incubation with nanoparticle-labeled T cells (A; right column, bottom row) or unlabeled T cells (A; right column, middle row). Further quantification indicates similar GzmB activity for labeled and unlabeled T cells following co-incubation with B16 F10 (B) or B16 OVA (C) cancer cells.

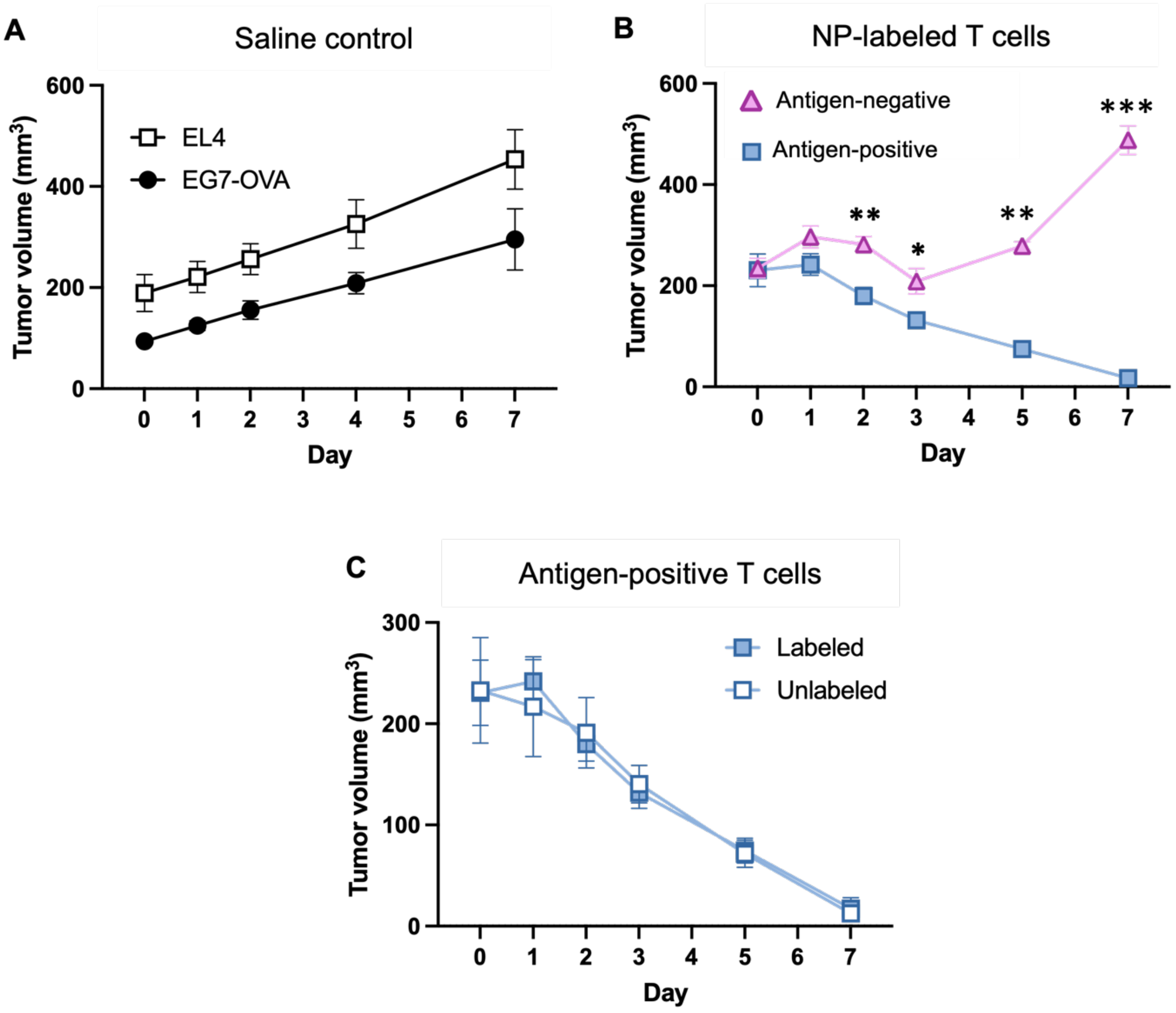

Figure S11. Caliper measurements of tumor volumes to validate the mouse model system and support US/PA imaging studies *in vivo*. All mice were sub-lethally lymphodepleted via x-ray irradiation prior to receiving saline injections or T cell injections. (A) Mice were inoculated (n = 3 per group) with EL4 tumors (open box) or EG7-OVA tumors (filled box) and received systemic injections of saline via the tail vein. Similar trends in tumor growth were observed and differences between groups were non-significant at all time points. For Day 7, statistical analysis was not possible – one mouse in the EG7-OVA group unexpectedly died before tumor measurements could be acquired (n = 2). (B) Tumor growth for mice (n = 4 per group) inoculated with EG7-OVA or

EL4 tumors. Both groups received NP-labeled T cells. The T cells were harvested from the spleens of OT-1 donor mice, meaning the T cell receptor specifically recognizes and targets EG7-OVA (antigen-positive; blue curve), but not EL4 (antigen-negative; pink curve). As expected, only the EG7-OVA mice respond to treatment with the NP-labeled OT-1 T cells. (C) Tumor growth for mice (n = 4 per group) inoculated with EG7-OVA tumors that received NP-labeled (filled box) or unlabeled T cells (open box). Similar response was observed between the labeled and unlabeled control, indicating similar efficacy *in vivo*, regardless of NP-labeling. Comparisons between groups at each timepoint were non-significant. Plotted values are the mean ± standard error of the mean. Data were analyzed with an unpaired student's T test comparing groups at each timepoint. * $p<0.05$; ** $p<0.01$; *** $p<0.001$. All data points without an asterisk are non-significant.

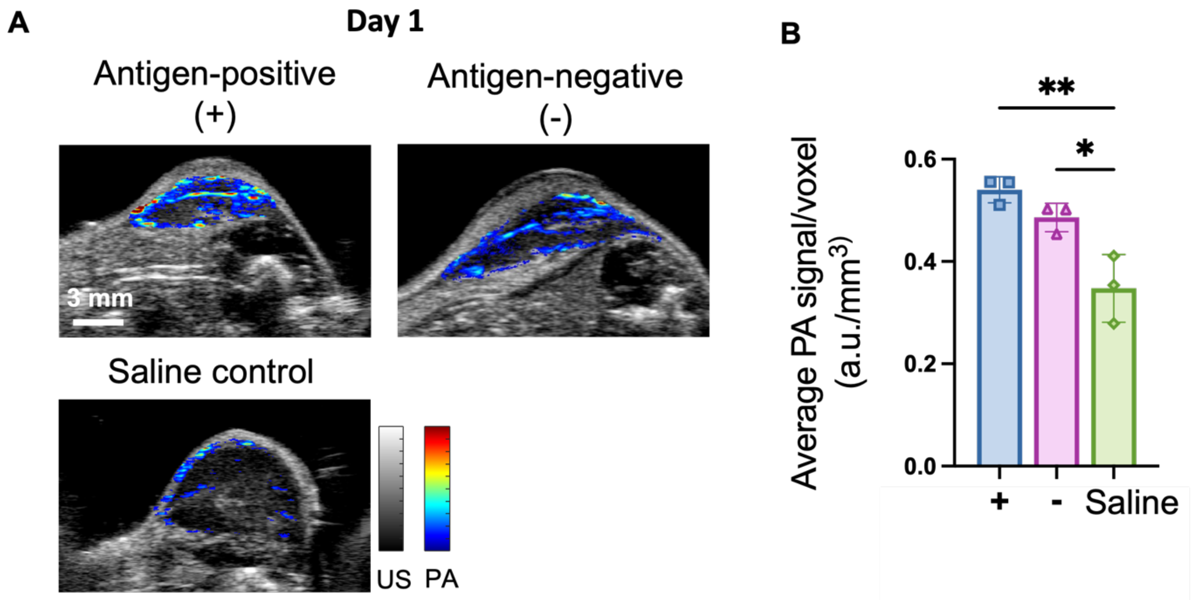

Figure S12. Verification that PA signal from labeled T cells is above background. (A) US/PA images were acquired 24 hours following systemic injection of labeled T cells or saline controls. Qualitatively, a clear increase in PA signal was observed for mice that received antigen-positive (+) or antigen-negative (-) NP-labeled T cells compared to the saline control, where minimal PA signal was observed within the tumor volume. (B) Quantitative analysis ($n = 3$ per group) confirmed a statistically significant increase in PA signal for mice that received labeled T cells over the background signal (saline control). Plotted values are the mean ± standard deviation. Data were analyzed with an unpaired student's T test comparing each group to the saline control. * $p<0.05$; ** $p<0.01$; *** $p<0.001$.

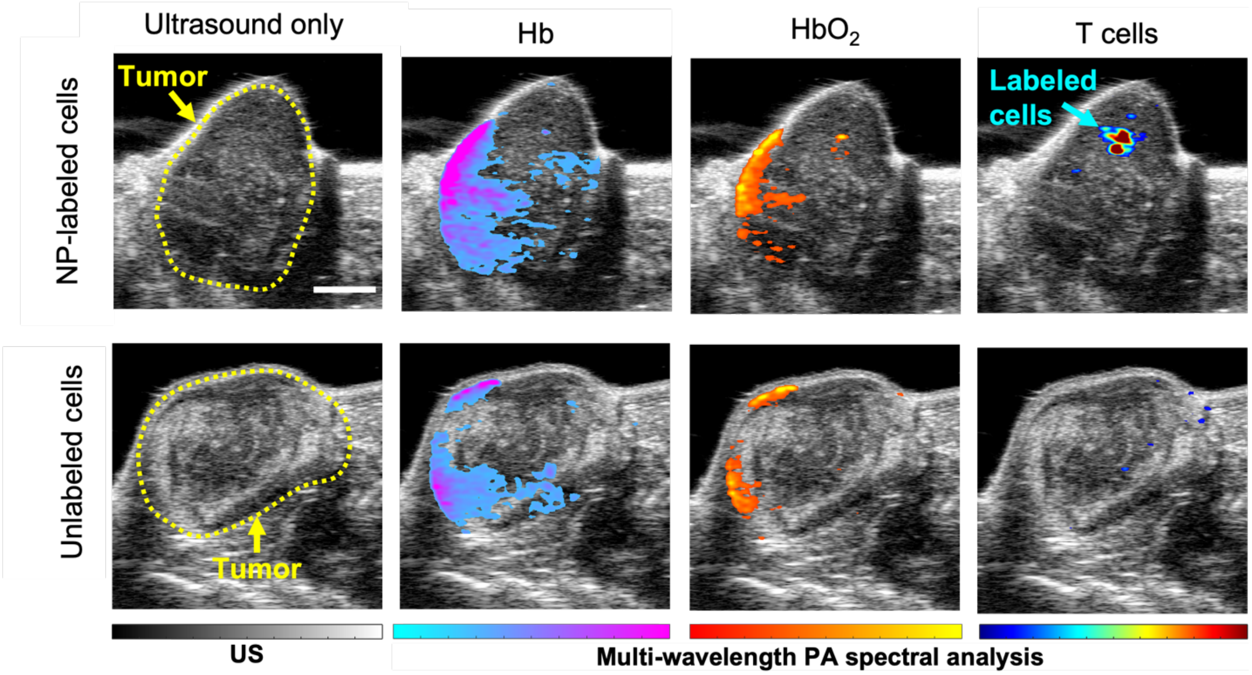

Figure S13. Imaging controls to develop spectral unmixing algorithm to distinguish blood biomarkers and labeled primary T cells *in vivo.* Mice received intratumoral injections of 100k NP-labeled T cells (OD = 5; top row) or 100k unlabeled T cells (bottom row), serving as the positive and negative imaging controls, respectively. Multi-wavelength PA datasets were acquired from 700 nm – 900 nm wavelength in 25 nm intervals (11 wavelengths total) and post-processed in MATLAB to evaluate crosstalk following spectral unmixing for deoxygenated hemoglobin (Hb) or oxygenated hemoglobin (HbO_2) and labeled T cells. Minimal crosstalk between absorbers was observed. In the positive imaging control (top row), the PA signal from labeled T cells was correctly localized to the isolated region of the injection. Alternatively, without T cell injection, i.e. the negative imaging control (bottom row), within the primary tumor, PA signal from Hb or HbO_2 was not incorrectly identified as labeled T cells. Scale bar = 2 mm.
